# Decoupling Plasmonic Hot Carrier from Thermal Catalysis via Electrode Engineering

**DOI:** 10.1021/acs.nanolett.4c01803

**Published:** 2024-07-08

**Authors:** Pandiaraj Sekar, Robert Bericat-Vadell, Yeersen Patehebieke, Peter Broqvist, Carl-Johan Wallentin, Mikaela Görlin, Jacinto Sá

**Affiliations:** † Department of Chemistry-Ångström, Physical Chemistry Division, Uppsala University, Uppsala 751 20, Sweden; ‡ Department of Chemistry and Molecular Biology, University of Gothenburg, Kemivägen 10, Gothenburg 412 58, Sweden; § Department of Chemistry-Ångström, Structural Chemistry Division, Uppsala University, Uppsala 751 20, Sweden; ∥ Institute of Physical Chemistry, Polish Academy of Sciences, Warsaw 01-224, Poland

**Keywords:** Energy filter, plasmonic hot carriers, single-electron transfer catalysis, reduced surface heat accumulation, photo electrocatalysis

## Abstract

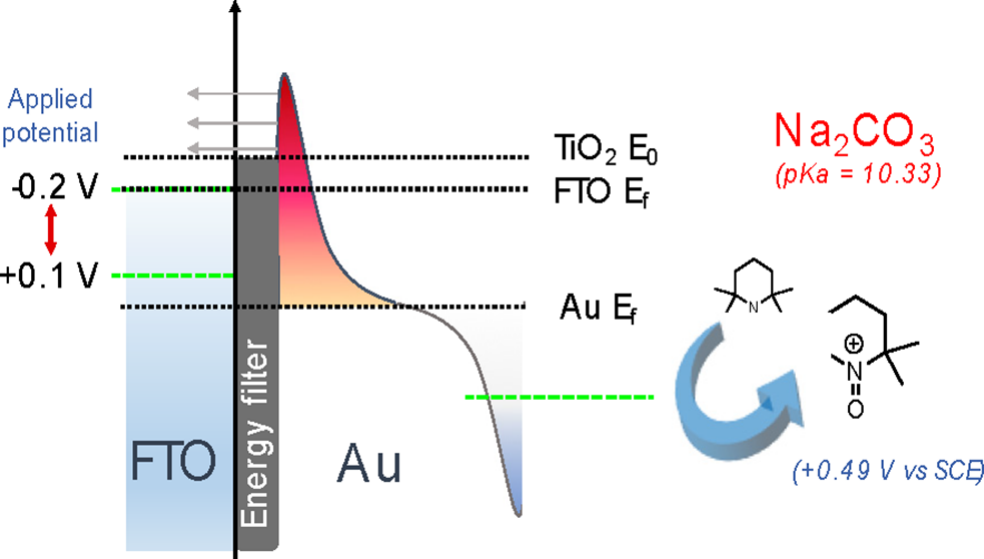

Increased attention has been directed toward generating nonequilibrium hot carriers resulting from the decay of collective electronic oscillations on metal known as surface plasmons. Despite numerous experimental endeavors, demonstrating hot carrier-mediated photocatalysis without a heating contribution has proven challenging, particularly for single electron transfer reactions where the thermal contribution is generally detrimental. An innovative engineering solution is proposed to enable single electron transfer reactions with plasmonics. It consists of a photoelectrode designed as an energy filter and photocatalysis performed with light function modulation instead of continuously. The photoelectrode, consisting of FTO/TiO_2_ amorphous (10 nm)/Au nanoparticles, with TiO_2_ acting as a step-shape energy filter to enhance hot electron extraction and charge-separated state lifetime. The extracted hot electrons were directed toward the counter electrode, while the hot holes performed a single electron transfer oxidation reaction. Light modulation prevented local heat accumulation, effectively decoupling hot carrier catalysis from the thermal contribution.

Surface plasmons, the collective oscillations of conduction electrons in metallic nanostructures, have become a fundamental elementary excitation in condensed matter and critical to numerous practical applications. Chemical reactions facilitated by plasmons often involve the participation of highly energetic charge carriers, commonly called “hot carriers”, and an augmented local temperature resulting from hot carriers recombination. In plasmonically excited metals, the distribution of charge carriers significantly diverges from the equilibrium Fermi–Dirac distributions, leading to an increased population of energetic charge carriers.^1,2^ These energetic charge carriers play a crucial role in promoting chemical reactions through various mechanisms, including direct reduction/oxidation of reactants,^3^ modulation of adsorption/desorption behavior of intermediates to lower the thermal activation energy barrier,^4^ enhancement of interfacial electron transfer,^5^ and mediation of catalyst chemical valency.^6^ Furthermore, the interaction between energetic carriers and the phonon modes of plasmonic metals can rapidly elevate the lattice temperature within a few picoseconds.^7^ This plasmon-induced local heating excites the vibrational transitions of reactants, thereby accelerating chemical reactions. This phenomenon, known as the thermal effect, is well described in classical transition state theory.^8^

Disentangling and weighting the contributions of hot carriers and thermal effects pose a notable challenge. To complicate this further, recent investigations indicate potential contributions from plasmon-induced near-field enhancement and photopotentials arising from the asymmetric accumulation of hot carriers.^9,10^ However, coupling of hot carriers mediated catalysis with thermal processes operates synergetically; in general, thermal effects harm single electron transfer (SET) reactions, which constitute the foundation of contemporary synthetic chemistry.^11^ This is because local heat opens the possibility for side reactions and introduces ambiguity to redox potentials, favoring double reduction/oxidation processes instead of radical formation via a SET process.

In plasmon-mediated photoelectrochemical reactions, the contribution of hot carriers and thermal effects can be accurately assessed by quantitatively analyzing the photocurrent response curves.^12,13^ Light-modulated reactions mediated solely by hot carriers should exhibit an immediate photocurrent response to light, with a characteristic spike and a square-like signal superimposing the square-wave light pulse.^14,15^ Conversely, the thermal contribution manifests as a gradual response to light, displaying a characteristic scaling with the square root of time (*t*^1/2^).^15,16^ Despite operating on different time scales, the thermal contribution rapidly challenges hot carrier processes (within ∼0.02 s)^14^ since heterogeneous catalytic turnover occurs in the milliseconds, if not longer, time scales.^17^ Consequently, it becomes imperative to design electrodes that extend the lifetimes of charge separation states and lessen local heat accumulation.

Herein, it is proposed to incorporate an energy filter between the electron collector and the plasmonic structure to improve charge separation, thus achieving extended lifetimes of hot carriers. This energy filter is an ultrathin insulating layer (in this case, a 10 nm amorphous TiO_2_ layer) between the two structures, characterized by a specific transmission function (), in this case a step-shaped  was used, which selectively collects carriers with energies (Ε) surpassing the filter energy threshold (Ε_0_). Amorphous TiO_2_ was selected as the insulating energy filter material because of suitable Ε_0_ and commercial availability. The FTO with the TiO_2_ amorphous (10 nm) is fabricated by NSG-Pilkington.

To test the energy filter concept hypothesis, an electrode consisting of FTO/TiO_2_ amorphous (10 nm)/Au nanoparticles (NPs) (from now on labeled as FTO/TiO_2_) of fluorinated tin oxide (FTO) glass and Au NPs are 4.6 and 5.0–5.2 eV,^18,19^ respectively. The energy filter consists of an amorphous TiO_2_ structure. The amorphous TiO_2_ work function energy edge (Ε_0_) is expected at 4.4–4.5 eV.^20,21^ Validation experiments with ultraviolet photoelectron spectroscopy gave a work function at 4.6 eV, corroborating the published literature. Cyclic voltammetry (CV) analysis revealed that the TiO_2_ layer is pinhole-free since it suppresses the FTO glass reaction with the electrolyte (see Supporting Information (SI) Figure S2).

The Au NPs were prepared via top-down metal deposition followed by annealing at 723 K for 30 min (details related to the fabrication can be found in SI). The annealing process narrowed the width and increased the intensity of the absorption peak, according to the UV–vis analysis shown in Figure S3. These changes indicate the confinement of the surface plasmon resonance, consistent with nanoparticle formation. This was corroborated by scanning electron microscopy (SEM) micrographs (Figure 1b and Figure S4) that revealed Au particles ranging from 10 to 20 nm after annealing that are uniformly distributed throughout the TiO_2_ surface. The UV–vis spectrum has a maximum absorption peak at 610 nm, corresponding to the maximum localized surface plasmon resonance (LSPR). The elemental analysis map (Figure S5) confirmed the presence of Au with an atomic abundance of ca. 0.22%. Note that elemental analysis with energy-dispersive X-ray detection at the SEM is a bulk technique probing about 1–3 μm deep. Thus, the atomic abundance reflects the atom concentration with the probing volume, not the surface, where all the gold is located.

**Figure 1:**
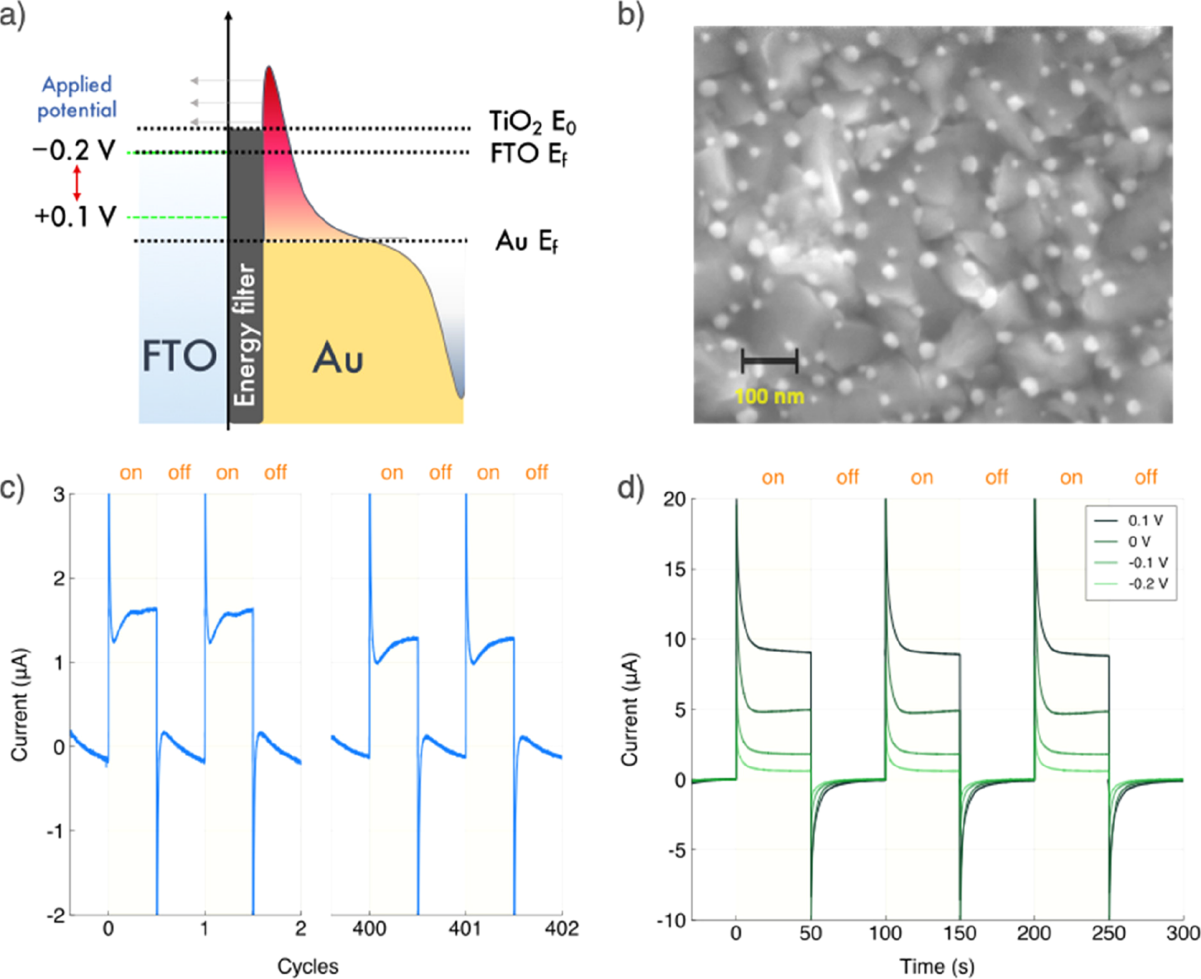
Plasmonic energy filter photoelectrode concept. (a) Schematic representation of the energy filter electrode concept, with the arrows indicating the foreseen electron transfer process. The hot holes that remain in Au NPs react with TEMPO according to Scheme S1. The applied potential operates solely on FTO Ε_*f*_, enabling regulation of electron back transfer process; (b) SEM image of Au NPs prepared via top-down approach on the energy filter electrode; (c) Photocurrent response during 12 h of oxidative addition of TEMPO to phenyl methylcarbamate reaction at 0.1 V vs Ag/AgCl under 633 nm CW illumination with a 20 mHz modulation, demonstrating system long-term stability. One cycle equates to 100 s (50 s on and 50 s off); and (d) Effect of applied potential in the FTO/TiO_2_/Au electrode photocurrent response associated with TEMPO reversible oxidation under 633 nm CW illumination with a 20 mHz modulation.

X-ray photoelectron spectroscopy (XPS) at the Au *4f* yielded a doublet related to a single species of Au with an Au *4f*_*7/2*_ peak at 83.7 eV ascribed to gold in a metallic state^22^ (see in SI Figure S6 after sample charging correction using C*1s* signal 284.6 eV). XPS analysis detected the presence of the ultrathin amorphous TiO_2_ layer with a characteristic Ti *2p*_*3/2*_ peak with a binding energy of 458.6 eV consistent with Ti^4+^ in the TiO_2_ lattice (Figure S7).^23^ CV analysis revealed the appearance of peaks associated with characteristic surface Au oxidation and reduction but no additional peaks, suggesting that Au NPs fabrication did not affect the quality of the TiO_2_ layer (Figure S8), i.e., the TiO_2_ layer remained pinhole-free.

The effectiveness of the energy filter in a photocatalytic process was evaluated with the 2,2,6,6-tetramethylpiperidine-1-oxyl (TEMPO) reversible oxidation. An electrode with FTO/mesoporous TiO_2_/Au was also fabricated for comparison. This electrode has been evaluated before and achieves charge separation via hot electron injection into the TiO_2_.^3^ CV analysis of the electrodes performed in the reaction mixture revealed no oxidation peak up to 0.2 V vs Ag/AgCl in the absence of light (Figures S9 and S10). Note that measurements were performed with a 633 nm continuous wave (CW) laser to excite the Au LSPR and avoid interband excitation.^24^ Moreover, the long excitation wavelength eliminates the possibility of direct substrate excitation.^25^

Measurements at 0.1 V vs Ag/AgCl showed current only when Au and TEMPO are present (Figures S11 and S12), strongly suggesting that it relates to plasmonic photoprocess. More importantly, the photocurrent response to the light function resembles the electrocatalysis current response to potential steps performed far from the formal potential of the electroactive group (*E*^*0’*^), as reported by Chidsey in his seminal work.^26^ Analogous to Chidsey’s potential step, at the start of a light switching step, there is initially a short current transient due to charging/discharging of the electrochemical double-layer, positive when the light is turned on and negative when the light is switched off.

The distinctiveness of the photocurrent response becomes even more apparent if one compares it with the FTO/mesoporous TiO_2_/Au response (Figure S13). The latter shows a relatively slow response to the light function without the sharp peak related to the double-layer charging and electron transfer reaction, which is consistent with a process having a concomitant contribution of hot carriers and heat. Excessive amounts of local heat significantly reduce the photocurrent intensity response, as confirmed by the nearly 10-fold decrease in photocurrent observed with the FTO/mesoporous TiO_2_/Au electrode compared with the energy filter electrode (Figure S13). However, the FTO/TiO_2_/Au photoresponse is not perfectly square, revealing that after the initial heat-free electron transfer, there is some heat accumulation. Yet, this is significantly less than with the FTO/mesoporous TiO_2_/Au electrode, governed by the Marcus-Gerischer model to separate the charges.

To further corroborate that the measured photocurrents are related to TEMPO oxidation, the oxidized TEMPO (TEMPO^+^, *E*^0^ = +0.49 V vs SCE)^27^ was used as an oxidant in the multisite proton-coupled electron transfer (PCET) reaction of 3-methylbut-2-enyl phenyl carbamate in the presence of Na_2_CO_3_ base (p*K*_a_ = 10.33),^28^ as published elsewhere (Scheme S1).^3^ The chronoamperometry data of the reaction is shown in Figure 1c, revealing a very stable photoresponse to the light function modulation. After a 12h reaction using a light modulation of 20 mHz (50 s on and 50 s off), a single product was isolated with a 53% yield, equating to a reaction rate of 88 nmol/h, comparable to what was reported previously.^3^ The ^1^H and ^13^C NMR (Figures S14 and S15) of the isolated compound corresponds to 3- phenyl-4-(2-((2,2,6,6-tetramethylpiperidin-1-yl)oxy)propan-2-yl)oxa-zolidin-2-one,^3,29^ confirming C–N bond formation via multisite PCET oxidative single electron transfer N-centered radicals.^30^

Energetic relationships for assessing hydrogen bond strengths typically rely on a thermodynamic cycle equating the observed bond dissociation free energy (BDFE) to the sum of energies needed for heterolytic bond breakage (*pK*_*a*_ value) and the one-electron oxidation of the conjugate base to a neutral nitrogen free radical (N·), along with the reduction of H^+^ to H· (redox potentials). The thermodynamic capacity of an oxidant/base to act as a formal H· acceptor can be described by an identical thermochemical cycle termed effective BDFE (denoted as “BDFE”), according to Mayer et al. (eq S1).^31^ Knowles^32^ argued that when the multisite PCET “BDFE” matches a specific bond BDFE, that bond undergoes selective homolysis even when weaker bonds are present. The substrate N–H BDFE ≈ 80.5 kcal/mol,^33^ and the estimated “BDFE” for our system is 80.35 kcal/mol, strongly supporting that TEMPO is oxidized and consequently utilized as the oxidant in the multisite PCET reaction.

Figure 1d shows the chronoamperometry data under light modulation at variable applied potential. The effect of the applied potential will be discussed later, but it is clear that the photoresponse increases when the applied potential is changed from −0.2 to 0.1 V vs Ag/AgCl.

The current response to the illumination step function provides a direct method to quantify hot carrier transfer kinetics. The Cottrell method is the most common model utilized to fit heterogeneous photoelectrocatalysis, which accounts for analyte diffusion. Briefly, in a simple redox event, such as TEMPO oxidation, under diffusion-controlled conditions, the measured current depends on the rate at which the analyte diffuses to the electrode, described by the Cottrell eq (eq S2). The plot of *i* vs *t*^–1/2^ (*i* and *t* are the measured current and time, respectively) provides a value of the collection constant for a given system (*k*), which is given from the slope.

The Cottrell fitting of the chronoamperometry data under different applied potentials is shown in Figure S16. The estimated collection constants are shown in Table S1. From the collection constants, the diffusion coefficients were estimated to be around 10^–12^ cm^2^/s, significantly smaller than the expected value for TEMPO in acetonitrile (10^–6^–10^–7^ cm^2^/s).^34^ The findings suggest that substrate diffusion does not affect the measured photocurrent responses. Hence, the photocurrent response can be fitted as an electrode-substrate interface with an immutable substrate concentration, i.e., the amount of TEMPO at the electrode is significantly higher than the amount consumed by the reaction. Under this consideration, one can adopt Chidsey’s approach to estimate hole transfer decay rates.^26^ The quality of the fittings is exemplified in SI Figure S17.

According to Chidsey,^26^ when current transients are slow enough to be accurately measured, potential-step experiments (mimicked in the present study by the light modulation step). The decay rate is estimated from the slope of the semilogarithmic plots of I vs t after the cell capacity response. The fitted values are shown in Table S1. The estimated values are around 0.4 s^–1^ independent of the applied potential, suggesting that the applied potential does not affect the hot hole transfer rate and its transfer mechanism. This signifies that the applied potential operates solely on the electron back transfer from FTO to Au NPs, which affects the number of hot holes available for the reaction. Still, the hot hole transfer process from Au to TEMPO is independent of the hot hole population.

In an energy filter concept, the energy filter material electrically disconnects the cold (FTO) and hot (Au plasmonic) carrier reservoirs. The energy filter barrier regulates the electron transmission until the reservoirs equilibrate, i.e., there is a saturation level. By modulating the energy level of the cold reservoir, the saturation level can be effectively manipulated. Since the reservoirs are electrically disconnected, the applied external bias effectively adjusts the rate of back-electron transfer, thereby augmenting the system’s overall efficiency through increased charge separation.

Figure 2a shows the photocurrent response’s dependence on light intensity. Increasing light intensity increases the photocurrent response. As with the applied potential, the photoresponse fitted with the Cottrell equation yielded collection constants with unreasonably low diffusion coefficients (Table S2). Therefore, the decay rates were once more fitted with the Chidsey approach.^26^ The estimated decay rates were between 0.5–0.6 s^–1^ (Table S2), similar to the values with different applied potentials but, more importantly, largely independent of the fluency used. The increase in fluency increases the population of reactive hot holes, but this does not affect the hot hole transfer process from Au to TEMPO, corroborating the findings with different applied potentials.

**Figure 2:**
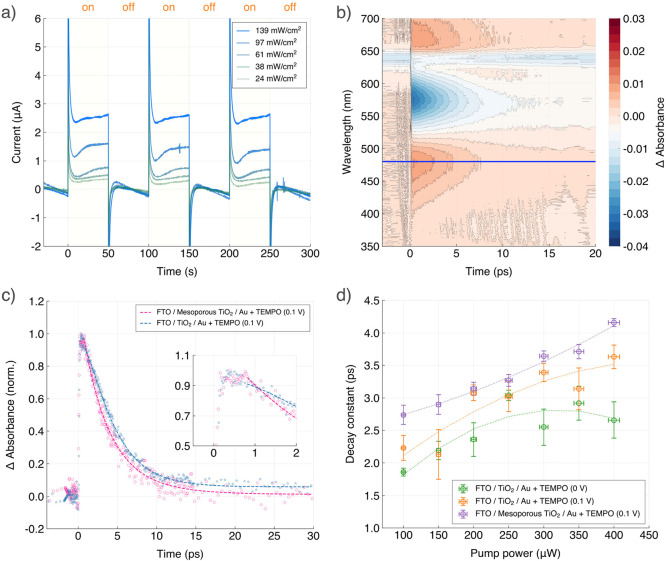
Plasmonic energy filter photoelectrode response to light intensity. (a) Effect of CW laser (633 nm) fluency in the electrode photocurrent response associated with TEMPO reversible oxidation at 0.1 V vs Ag/AgCl applied potential with a 20 mHz modulation; (b) TAS contour map after excitation of the energy filter electrode at 633 nm at 0.1 V vs Ag/AgCl applied potential; (c) Comparison of the kinetic traces extracted at the maximum of the positive winglet between energy filter and conventional (with mesoporous TiO_2_) electrodes under the same laser pump power (250 μW), excitation wavelength and applied potential; (d) Effect of the pump laser power in the electronphonon scattering lifetime.

To understand the independent behavior of the photoresponse to applied potential and light intensity, ultrafast transient absorption spectroscopy (TAS) measurements were performed.^35^Figure 2b shows a contour plot of FTO/TiO_2_/Au at 0.1 V vs Ag/AgCl in the reaction solution after excitation at 633 nm. The map shows the characteristic Au NPs bleach around the excitation wavelength and a positive winglet to the blue of the bleach resultant from the photoinduced broadening.^36,37^ A similar winglet appears to the bleach’s blue but is only partially detected due to the probe pulse energy range.

Figure 2c presents the kinetic traces extracted at the maximum of the winglet to the blue of the bleach (exemplified by the blue line in the contour map in Figure 2b) for the FTO/TiO_2_/Au and FTO/mesoporous TiO_2_/Au. There is a noticeable difference in the signal shape between the two samples. While the FTO/mesoporous TiO_2_/Au show the expected fast rise followed by a smooth exponential decay, consistent with fast electron injection into TiO_2_ and relaxation of the less energetic electrons through electron–phonon (*e-ph*) scattering,^38^ the FTO/TiO_2_/Au had a slower rising edge that is better depicted in the figure insert, consistent with a slower electron transfer. The implications are that in the case of the FTO/mesoporous TiO_2_/Au, the injection occurs as the hot carrier multiplication is taking place, while in the FTO/TiO_2_/Au, the electrons are transferred after the multiplication step is nearly completed, which can take up to 500 fs.^39^

To test this hypothesis, the *e-ph* lifetimes were estimated from TAS measurements under different pump pulse fluencies (Figure 2d). It has been demonstrated that *e-ph* lifetime is highly sensitive to the amount of hot carriers participating in the plasmon resonance process.^38^ The hot electrons’ average temperature on Au NPs can be estimated from excitation power dependence electron–phonon relaxation time (τ_*e*-*ph*_) according to equation S3.^40^Figure S18 shows the kinetic traces extracted at the maximum of the winglet to the blue of the Au NPs supported on glass. The fitted τ_*e*-*ph*_ and corresponding average electron temperatures are shown in Table S3. Except for the very low laser power excitation, the average electronic temperature is ca. 1000 K, which is within a factor of 2 of simple estimates based on the extended two-temperature model but consistent with what has been reported for Au-supported on solid substrates.^41^ Noticeably, the electronic temperature on Au NPs saturates, but the overall signal intensity (amplitude) increases with increased laser excitation power. This suggests that after reaching the saturation temperature, the increase in laser excitation power results in solely the number of hot carriers, not their energy.

The difference between the dynamics of the energy filter concept (FTO/TiO_2_/Au) and Schottky junction (FTO/mesoporous TiO_2_/Au sample) was noticeable from the onset of the data analysis. While in the case of the FTO/mesoporous TiO_2_/Au sample, the τ_*e*-*ph*_ was extracted from the first exponential decay, the FTO/TiO_2_/Au sample, since the transfer happens at a longer time scale, required the use of Sun’s approach to remove the contribution of the nonthermalizd electron distribution.^42^ The fitting procedure is described in SI and illustrated in Figure S1. The need for distinct procedures to treat ultrafast dynamics denotes the involvement of different charge transfer mechanisms, consistent with the various theoretical frameworks underpinning the electron transfer process for each electrode.

The FTO/mesoporous TiO_2_/Au sample, the τ_*e*-*ph*_ increases linearly with the increased pump pulse fluence (Figure 2d), consistent with previous results.^39,40^ Those results show that increased laser fluence increases the amount of hot electrons in the resonance that have sufficient energy to overcome the Au-TiO_2_ Schottky barrier (ca. 1 eV).^43,44^ No carrier multiplication occurs since the injection occurs immediately after forming the first hot carrier population. Consequently, no saturation is observed because the available excitable electrons dwarf the number of photons. As laser fluency increases, more carriers are formed. Still, due to the high Schottky barrier, only a small percentage is injected, effectively increasing the τ_*e*-*ph*_ with increased fluency.

In the case of the FTO/TiO_2_/Au sample, the transfer happens as the carrier multiplication is taking place.^41^ A non-Fermi–Dirac distribution of nonthermalized carriers characterizes this period. This is when the number of transferable electrons reaches a maximum. Increased laser fluency leads to an increase τ_*e*-*ph*_. Still, since the hottest electrons are not immediately transferred, like in the case of the Schottky junction, they undergo multiplication, reaching an electronic temperature saturation similar to what one observed with the Au NPs on glass. Also, here, a further increase in laser excitation power increases overall signal intensity (amplitude). This suggests that after reaching the saturation temperature, the rise in laser excitation power results in solely the number of hot carriers, not their energy, effectively increasing the proportion of injectable carriers that can be transferred across the energy filter carrier. Finally, it is perceptible that the applied potential does not significantly affect the energy filter sample τ_*e*-*ph*_, consistent with the photocurrent responses, corroborates that the applied potential operates solely on reducing the back-electron transfer from FTO to Au that occurs on a longer time scale.

In summary, an original engineering solution is proposed to decouple the hot carrier catalysis from photothermal catalysis, consisting of a photoelectrode designed as an energy filter and photoelectrocatalysis performed with light function modulation instead of continuously. Photocurrent response to light modulation reveals that hot carrier transfer occurs without significant interference of local heat. The extracted decay rates suggest that hot hole transferrence is independent of the hot hole population, indicating that the hot holes act as individual charges, which is critical for radical-mediated photoredox catalysis. The proposed approach offers a pathway to decouple hot carrier-mediated catalysis from the photothermal, which has hindered the successful exploitation of plasmonic hot carriers and instigated significant controversy within the research community.

## Supporting Information Available

Additional experimental details, materials, methods, mathematical formulas, reaction schemes, and theoretical support for the findings. It also includes additional supporting data, including characterization (e.g., microscopy, electrochemistry, X-ray photoelectron spectroscopy, and ultrafast spectroscopy) and catalytic data with NMR support for product formation and treatment of the photo electrocatalytic response to light modulation. (PDF)

## References

[ref1] Linic S.; Aslam U.; Boerigter C.; Morabito M. Photochemical transformations on plasmonic metal nanoparticles. Nat. Mater. 2015, 14, 567–576. 10.1038/nmat4281. 25990912

[ref2] Reddy H.; Wang K.; Kudyshev Z.; Zhu L.; Yan S.; Vezzoli A.; Higgins S. J.; Gavini V.; Boltasseva A.; Reddy P.; Shalaev V. M.; Meyhofer E. Determining plasmonic hot-carrier energy distributions via single-molecule transport measurements. Science 2020, 369, 423–426. 10.1126/science.abb3457. 32499398

[ref3] Bericat-Vadell R.; Sekar P.; Patehebieke Y.; Zou X.; Kaul N.; Broqvist P.; Lindblad R.; Lindblad A.; Arkhypchuk A.; Walletin C.-J.; Sá J. Single-electron transfer reactions on surface-modified gold plasmons. Mater. Today Chem. 2023, 34, 101783 10.1016/j.mtchem.2023.101783.

[ref4] Robatjazi H.; Bao J. L.; Zhang M.; Zhou L.; Christopher P.; Carter E. A.; Nordlander P.; Halas N. J. Plasmon-driven carbon-fluorine (C(sp3)–F) bond activation with mechanistic insights into hot-carrier-mediated pathways. Nat. Catal. 2020, 3, 564–573. 10.1038/s41929-020-0466-5.

[ref5] Liu G.; Li P.; Zhao G.; Wang X.; Kong J.; Liu H.; Zhang H.; Chang K.; Meng X.; Kako T.; Ye J. Promoting active species generation by plasmon-induced hot-electron excitation for efficient electrocatalytic oxygen evolution. J. Am. Chem. Soc. 2016, 138, 9128–9136. 10.1021/jacs.6b05190. 27380539

[ref6] Marimuthu A.; Zhang J.; Linic S. Tuning selectivity in propylene epoxidation by plasmon mediated photo-switching of Cu oxidation state. Science 2013, 339, 1590–1593. 10.1126/science.1231631. 23539599

[ref7] Inouye H.; Tanaka K.; Tanahashi I.; Hirao K. Ultrafast dynamics of nonequilibrium electrons in a gold nanoparticle system. Phys. Rev. B 1998, 57, 11334–11340. 10.1103/PhysRevB.57.11334.

[ref8] Sivan Y.; Un I. W.; Dubi Y. Assistance of metal nanoparticles in photocatalysis - nothing more than a classical heat source. Faraday Discuss. 2019, 214, 215–233. 10.1039/C8FD00147B. 30849158

[ref9] Wilson A. J.; Jain P. K. Light-Induced Voltages in Catalysis by Plasmonic Nanostructures. Acc. Chem. Res. 2020, 53, 1773–1781. 10.1021/acs.accounts.0c00378. 32786334

[ref10] Wilson A. J.; Mohan V.; Jain P. K. Mechanistic Understanding of Plasmon-Enhanced Electrochemistry. J. Phys. Chem. C 2019, 123, 29360–29369. 10.1021/acs.jpcc.9b10473.

[ref11] Mennen S. M.; Alhambra C.; Allen C. L.; Barberis M.; Berritt S.; Brandt T. A.; Campbell A. D.; Castañón J.; Cherney A. H.; Christensen M.; et al. The Evolution of High-Throughput Experimentation in Pharmaceutical Development and Perspectives on the Future. Org. Process Res. Dev. 2019, 23, 1213–1242. 10.1021/acs.oprd.9b00140.

[ref12] Ou W.; Zhou B.; Shen J.; Lo T. W.; Lei D.; Li S.; Zhong J.; Li Y. Y.; Lu J. Thermal and nonthermal effects in plasmon-mediated electrochemistry at nanostructured Ag electrodes. Angew. Chem., Int. Ed. 2020, 59, 6790–6793. 10.1002/anie.202001152. 32040261

[ref13] Zhan C.; Liu B.-W.; Huang Y.-F.; Hu S.; Ren B.; Moskovits M.; Tian Z.-Q. Disentangling charge carrier from photothermal effects in plasmonic metal nanostructures. Nat. Commun. 2019, 10, 2671 10.1038/s41467-019-10771-3. 31209216 PMC6572789

[ref14] Ou W.; Fan Y.; Shen J.; Xu Y.; Huang D.; Zhou B.; Lo T. W.; Li S.; Li Y. Y.; Lei D.; Lu J. Plasmoelectric Potential in Plasmon-Mediated Electrochemistry. Nano Lett. 2022, 22, 8397–8495. 10.1021/acs.nanolett.2c01035. 36190454

[ref15] Bagnall A. J.; Ganguli S.; Sekretareva A. Hot or Not? Reassessing Mechanisms of Photocurrent Generation in Plasmon-Enhanced Electrocatalysis. Angew. Chem., Int. Ed. 2024, 63, e202314352 10.1002/anie.202314352. 38009712

[ref16] Maley M.; Hill J. W.; Saha P.; Walmsley J. D.; Hill C. M. The Role of heating in the Electrochemical Response of Plasmonic Nanostructures under Illumination. J. Phys. Chem. C 2019, 123, 12390–12399. 10.1021/acs.jpcc.9b01479.

[ref17] Ardagh M. A.; Abdelrahman O. A.; Dauenhauer P. J. Principles of Dynamic Heterogeneous Catalysis: Surface Resonance and Turnover Frequency Response. ACS Catal. 2019, 9, 6929–6937. 10.1021/acscatal.9b01606.

[ref18] Michaelson H. B. The work function of the elements and its periodicity. J. Appl. Phys. 1977, 48, 4729–4733. 10.1063/1.323539.

[ref19] Khoa N. T.; Kim S. W.; Yoo D.-H.; Kim E. J.; Hahn S. H. Size-dependent work function and catalytic performance of gold nanoparticles decorated graphene oxide sheets. J. Appl. Catal. A 2014, 469, 159–164. 10.1016/j.apcata.2013.08.046.

[ref20] Lichterman M. F.; Hu S.; Richter M. H.; Crumlin E. J.; Axnanda S.; Favaro M.; Drisdell W.; Hussain Z.; Mayer T.; Brunschwig B. S.; et al. Direct Observation of the Energetics at a Semiconductor/Liquid Junction by Operando X-Ray Photoelectron Spectroscopy. Energy Environ. Sci. 2015, 8, 2409–2416. 10.1039/C5EE01014D.

[ref21] Feng G.; Hu M.; Yuan S.; Nan J.; Zeng H. Hydrogenated Amorphous TiO2–x and Its High Visible Light Photoactivity. Nanomaterials 2021, 11, 2801 10.3390/nano11112801. 34835567 PMC8625909

[ref22] Battistoni C.; Mattogno G.; Zanoni R.; Naldini L. Characterisation of some gold clusters by X-ray photoelectron spectroscopy. J. Electron Spectrosc. Relat. Phenom. 1982, 28, 23–31. 10.1016/0368-2048(82)80014-5.

[ref23] Bharti B.; Kumar S.; Lee H.-N.; Kumar R. Formation of oxygen vacancies and Ti3+ state in TiO2 thin film and enhanced optical properties by air plasma treatment. Scie. Rep. 2016, 6, 32355 10.1038/srep32355. PMC5004114 27572095

[ref24] Lyu P.; Espinoza R.; Nguyen S. C. Photocatalysis of Metallic Nanoparticles: Interband vs Intraband Induced Mechanisms. J. Phys. Chem. C 2023, 127, 15685–15698. 10.1021/acs.jpcc.3c04436. PMC10440817 37609384

[ref25] Falbo E.; Fusè M.; Lazzari F.; Mancini G.; Barone V. Integration of Quantum Chemistry, Statistical Mechanics, and Artificial Intelligence for Computational Spectroscopy: The UV–Vis Spectrum of TEMPO Radical in Different Solvents. J. Chem. Theory Comput. 2022, 18, 6203–6216. 10.1021/acs.jctc.2c00654. 36166322 PMC9558374

[ref26] Chidsey C. E. D. Free Energy and Temperature Dependence of Electron Transfer at the Metal-Electrolyte Interface. Science 1991, 251, 919–922. 10.1126/science.251.4996.919. 17847385

[ref27] Nutting J. E.; Rafiee M.; Stahl S. S. Tetramethylpiperidine N-Oxyl (TEMPO), Phthalimide N-Oxyl (PINO), and Related N-Oxyl Species: Electrochemical Properties and Their Use in Electrocatalytic Reactions. Chem. Rev. 2018, 118, 4834–4885. 10.1021/acs.chemrev.7b00763. 29707945 PMC6284524

[ref28] Choi G. J.; Knowles R. R. Catalytic Alkene Carboaminations Enabled by Oxidative Proton-Coupled Electron Transfer. J. Am. Chem. Soc. 2015, 137, 9226–9229. 10.1021/jacs.5b05377. 26166022 PMC4643263

[ref29] Xu F.; Zhu L.; Zhu S.; Yan X.; Xu H. C. Electrochemical intramolecular aminooxygenation of unactivated alkenes. Chem.—Eur. J. 2014, 20, 12740–12544. 10.1002/chem.201404078. 25145684

[ref30] Choi G. J.; Zhu Q.; Miller D. C.; Gu C. J.; Knowles R. R. Catalytic alkylation of remote C-H bonds enabled by proton-coupled electron transfer. Nature 2016, 539, 268–271. 10.1038/nature19811. 27732585 PMC5704892

[ref31] Warren J. J.; Tronic T. A.; Mayer J. M. Thermochemistry of proton-coupled electron transfer reagents and its implications. Chem. Rev. 2010, 110, 6961–7001. 10.1021/cr100085k. 20925411 PMC3006073

[ref32] Gentry E. C.; Knowles R. R. Synthetic Applications of Proton-Coupled Electron Transfer. Acc. Chem. Res. 2016, 49, 1546–1556. 10.1021/acs.accounts.6b00272. 27472068 PMC5102158

[ref33] Resa S.; Millán A.; Fuentes N.; Crovetto L.; Luisa Marcos M.; Lezama L.; Choquesillo-Lazarte D.; Blanco V.; Campaña A. G.; Cárdenas D. J.; Cuerva J. M. O–H and (CO)N–H bond weakening by coordination to Fe(II). Dalton Trans. 2019, 48, 2179–2189. 10.1039/C8DT04689A. 30672945

[ref34] Zhu F.; Zou Y.; Hua L.; Peng X.; Zhang W. Redox potential regulated by electrolyte concentration: A case study of electrochemical oxidation of 2,2,6,6-tetramethyl piperidine-1-oxyl. Electrochem. Commun. 2022, 142, 107374 10.1016/j.elecom.2022.107374.

[ref35] Furube A.; Du L.; Hara K.; Katoh R.; Tachiya M. Ultrafast plasmon-induced electron transfer from gold nanodots into TiO2 nanoparticles. J. Am. Chem. Soc. 2007, 129, 14852–14853. 10.1021/ja076134v. 17994750

[ref36] van Turnhout L.; Hattori Y.; Meng J.; Zheng K.; Sa J. Direct observation of a plasmon-induced hot electron flow in a multimetallic nanostructure. Nano Lett. 2020, 20, 8220–8228. 10.1021/acs.nanolett.0c03344. 33095592 PMC7662917

[ref37] Tagliabue G.; DuChene J. S.; Abdellah M.; Habib A.; Gosztola D. J.; Hattori Y.; Cheng W.-H.; Zheng K.; Canton S. E.; Sundararaman R.; Sá J.; Atwater H. A. Ultrafast Hot-Hole Injection Modifies Hot-Electron Dynamics in Au/p-GaN Heterostructures. Nat. Mater. 2020, 19, 1312–1318. 10.1038/s41563-020-0737-1. 32719510

[ref38] Hattori Y.; Gutierrez Alvarez S.; Meng J.; Zheng K.; Sa J. Role of the metal oxide electron acceptor on gold-plasmon hot-carrier dynamics and its implication to photocatalysis and photovoltaics. ACS Appl. Nano Mater. 2021, 4, 2052–2060. 10.1021/acsanm.0c03358.

[ref39] Heilpern T.; Manjare M.; Govorov A. O.; Wiederrecht G. P.; Gray S. K.; Harutyunyan H. Determination of hot carrier energy distribuitions from inversion of ultrafast pump-probe reflectivity measurements. Nat. Commun. 2018, 9, 1853 10.1038/s41467-018-04289-3. 29748626 PMC5945638

[ref40] Hodak J. H.; Henglein A.; Hartland G. V. Photophysics of Nanometer Sized Metal Particles: Electron–Phonon Coupling and Coherent Excitation of Breathing Vibrational Modes. J. Phys. Chem. B 2000, 104, 9954–9965. 10.1021/jp002256x.

[ref41] Conforti M.; Della Valle G. Derivation of third-order nonlinear susceptibility of thin metal films as a delayed optical response. Phys. Rev. B 2012, 85, 245423 10.1103/PhysRevB.85.245423.

[ref42] Sun C.-K.; Vallée F.; Acioli L. H.; Ippen E. P.; Fujimoto J. G. Femtosecond-tunable measurement of electron thermalization in gold. Phys. Rev. B 1994, 50, 15337–15348. 10.1103/PhysRevB.50.15337. 9975886

[ref43] Szydlo N.; Poirier R. I-V. and C-V Chracteritstics of Au/TiO2 Schottky diodes. J. Appl. Phys. 1980, 51, 3310–3312. 10.1063/1.328037.

[ref44] Sun Z.; Fang Y. Electrical tuning effect for Schottky barrier and hot-electron harvest in a plasmonic Au/TiO2 nanostructure. Scie. Rep 2021, 11, 338 10.1038/s41598-020-79746-5. PMC7801507 33432085

