# Supplementary material for: Decoupling Plasmonic Hot Carrier from Thermal Catalysis via Electrode Engineering
Source: Nano Lett. 2024 Jul 8;24(28):8619–25. doi: 10.1021/acs.nanolett.4c01803 (PMC11261604; doi:10.1021/acs.nanolett.4c01803)
Supplement: Supplementary file 1 — nl4c01803_si_001.pdf [file nl4c01803_si_001.pdf]

Supplementary information

Decoupling plasmonic hot carrier from thermal catalysis via electrode engineering

Pandiaraj Sekar[1#], Robert Bericat-Vadell[1#], Yeersen Patehebieke[2], Peter Bröqvist[3], Carl-Johan Wallentin[2], Mikaela Görlin[3] and Jacinto Sá[1,4*]

[1] Department of Chemistry-Ångström, Physical Chemistry division, Uppsala University, 751 20 Uppsala, Sweden.

[2] Department of Chemistry and Molecular Biology, University of Gothenburg, Kemivägen 10, 412 58 Gothenburg, Sweden.

[3] Department of Chemistry-Ångström, Structural Chemistry division, Uppsala University, 751 20 Uppsala, Sweden.

[4] Institute of Physical Chemistry, Polish Academy of Sciences, 01-224 Warsaw, Poland.

# equal contribution

* jacinto.sa@kemi.uu.se

EXPERIMENTAL & METHODS

Preparation of samples

Fabrication of the energy-filter electrode with Au NPs:

NSG-Pilkington fabricated FTO glass with 10 nm amorphous TiO_2 with a conduction band edge (E_0) at 4.4 eV.[1,2] Initially, the TiO_2 coated films were cleaned with Helmax (2%) solution in water for 30 mins under bath sonication, followed by washing with deionized water and dried under N_2 gas, to ensure the glasses were free from any impurities. Finally, the films were subjected to Ozone cleaning for 15 min to get rid off any organic impurities from the surface. The precleaned TiO_2 films were directly placed in the chamber of the evaporator (Physical vapour deposition /LEICA Instruments), and 2 nm of gold was deposited on the surface of the films. The Au nanopartilces deposited films were annealed at 450 °C for 30 minutes in a muffle furnace. After cooling down to the ambient temperature the films were directly used for the studies. The reproducibility of Au NPs deposition was examined by the UV-Vis absorption peak of the Au localised plasmon resonance and confirmed by X-ray photoelectron spectroscopy (XPS).

For comparison purposes a sample with mesoporous TiO_2 instead of amorphous layer was also prepared using Solaronix paste[3] diluted (150 mg paste/ml ethanol) followed by annealing at 500 °C for 30 minutes. Further gold deposition was carried out on the films using the same protocol mentioned in the earlier. The sample extracts the charge *via* electron injection, as commonly done. The subsequent sets to make the Au NPs were kept the same as the sample for the energy filter.

Characterisation of films

UV-Vis measurements:

The UV-Vis spectra were collected using a Cary 5000 UV-VIS-NIR spectrophotometer.

Cyclic voltametry measurements:

The cyclic voltommetry (CV) experiments were carried out in a 1.5 mL photoelectrochemical spectroscopy cell from Redox.me. The tests were performed with single films (the cell can accommodate two films) in a three-electrode configuration with Ag/Ag ion (non aqueous reference) and Pt as reference and counter electrodes, respectively. The CV was carried out at a potential between -1.5 to 1.2 V (Vs Ag/Ag ion) with the scan rate of 100 mV/s.

Scanning electron micrsocpy (SEM) measurements:

SEM was performed using a Zeiss Merlin microscope (Myfab, Ångström Laboratory). The acceleration voltage was 5 or 10 kV, and the working distance was between 5-7 mm.

X-ray photoelectron spectrsocpy (XPS) measurements:

The XPS measurements were recorded using Al Ka (1487 eV) radiation and a Quantera II spectrometer from Physical Electronics (Myfab, Ångström Laboratory). The data were fitted using the doublet separation provided by the NIST XPS database.[4] The charging was corrected using the advantageous C 1s peak, set to 284.6 eV.

<u>Catalytic testing</u>

The chronoamperometry experiments were carried out in a 1.5 mL photoelectrochemical spectroscopy cell from Redox.me. The tests were performed with single films (the cell can accommodate two films) in a three-electrode configuration with Ag/Ag ion (non aqueous reference) and Pt as reference and counter electrodes, respectively. The electrolyte was prepared by Bu_4NBF_4 (0.1 mol) in acetonitrile (10 ml) solvent followed by sonicating for few minutes. The photoelectrode exposed area to light is 0.95 cm^2. Plasmonic excitation was done with a 633 nm laser with a maximum power of 139 mW/cm^2 , illumination (on) and without light (off) for (50 seconds) time intervals.

TEMPO reversible oxidation:

The TEMPO oxidation were performed using chronoamperomtetry technique by applying a constant potentials for an hour with the same elecrode film and the configuration. Potential optimization experiments were carried out in a similar manner by applying the various potentials (-0.2, -0.1, 0.0 and 0.1 V (Vs Ag/Ag ion) for an hour. Controlled experiments were done with and without TEMPO to ensure the photocurrent generation related to the TEMPO oxidation.

Oxidative addition of TEMPO to phenyl methylcarbamate:

The photoelectrocatalysis of pheny methylcarbamate with TEMPO were done in a large cell (volume of 15 mL) photoelectrochemical spectroscopy cell from Redox.me. Initially, the substrate (0.1 mmol, 1 equiv), TEMPO (0.2 mmol, 2 equiv), Bu_4NBF_4 (1.0 mmol) and Na_2CO_3

(0.1 mmol) were dispersed in a suitable solvent mixture (Acetonitrile (9.5 mL) + H_2O (0.5 mL)) a clean beaker and sonicated for few minutes. Further, the reactant mixture was transferred to an air-tightened photoelectrochemical cell. Before the experiment, the cell was purged with N_2 gas for 15 mins. The tests were performed with single films (the cell can accommodate two films) in a three-electrode configuration with Ag/Ag ion (non-aqueous reference) and Pt as a reference and counter electrodes, respectively. The photoelectrode exposed area to light is 0.95 cm^2.

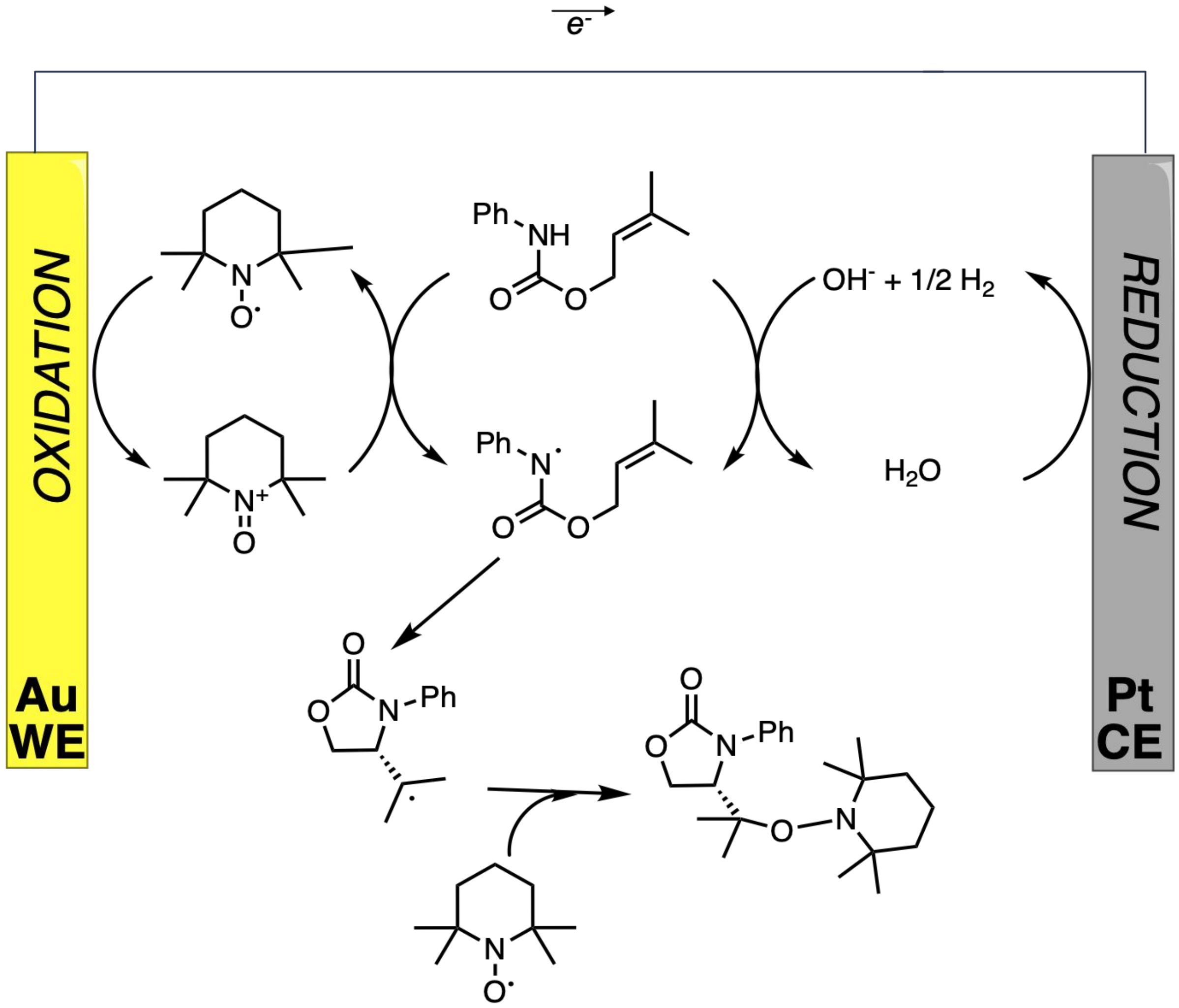

Scheme S1. Schematic representation of the aminooxygenation of activated alkenes with TEMPO.

The oxidation experiments were carried out using the chronoamperometric method by applying a constant potential of 0.1 V (vs Ag/Ag ion (non-aqueous reference)) for 12 h. During the measurements, the laser was illuminated through the cell's optical window and under on and off at a constant time interval (50 seconds). The reaction was monitored by thin-layer chromatography and ^{1}H-NMR techniques. After the reaction, the reaction mixture was poured into H_2O (25 mL) and the aqueous layer was extracted with EtOAc (3 x 25 mL). The organic

layers combined and washed with brine, dried over Na_2SO_4, filtered and concentrated under reduced pressure. The crude was purified by using flash silica gel column chromatography (eluent Dichloromethane : Hexane = 1:1) to get the title compound. ^{1}H and ^{13}C NMR spectroscopy was performed using $CDCl_3$ as a solvent.

Effective bond dissociation free energy ("BDFE") expression[5]:

$$"BDFE"(kcal/mol) = 1.37\, pKa + 23.06\, E^0 + C_{G,sol} \quad eq.\ S1$$

$C_{G,sol}$ (acetonitrile) = 54.9 kcal/mol

Chronoamperometry data fitting with Cottrell equation[6]:

$$i = \frac{nFAc_j^0\sqrt{D_j}}{\sqrt{\pi t}} \qquad eq. S2$$

i = current, in A

j = analyte (TEMPO)

n = number of electrons to oxidize one molecule TEMPO (n= 1)

F = Faraday constant (96485 C/mol)

A = area of the (planar) electrode in cm^2

c_j^0 = initial concentration of the reducible analyte in mol/cm^3

D_j = diffusion coefficient for TEMPO in acetonitrile in cm^2/s

t = time in s.

Ultrafast spectroscopy

Transient absorption spectroscopy (TAS)

A 40-fs pulsed laser with a 3 kHz repetition rate was generated through the Libra Ultrafast Amplifier System designed by Coherent. An optical parametric oscillator (TOPAS- prime, Light Conversion) generated the excitation beam. The signals were detected with a UV-NIR detector and a Newport MS260i spectrograph with interchangeable gratings. The fundamental laser (probe, 795 nm) passes through the delay stage (1-2 fs step size) and is focused in a CaF_2 optical window to generate visible light from 400 to 750 nm. The instrument response function obtained for our system is ca. 95 fs.

The full photoelectrochemical spectroscopy cell is placed in the sample holder, connected to the potentiostat and with its working electrode (the sample) facing the laser source. Both the pump and probe are overlapped on the internal electrode surface and then the time zero is found. To regualate the intensity of the pump, a variable neutral density filter placed before the cell is used. The power of the pump is measured using a LM-2 Vis smiconductor power sensor by Coherent, and it is ensured that the measured power does not present a standard deviation above 2%. For the power dependence measurements 7 different intensities where selected (100 μW, 150 μW, 200 μW, 250 μW, 300 μW, 350 μW, 400 μW) with a pump diameter at the sample of approximately 260 μm. For each measurement, at least 4 scans are performed and subsequently averaged to decrease the noise.

The fitting of the transient absorbance signal at the plasmonic wing were performed using the model developed by Sun et al. (see equation (8) in reference[7]) instead of using a sum of exponentials as it is commonly done. As can be seen in Fig. S1, for the energy filter sample an exponential function fails to fit the sigmoidal-like shape of the obtained data. This is because, unlike in other cases, here the contribution of the non-thermalized electron population (yellow dotted line in S1) is not neglectable in the picosecond time sacale and, therefore, its dynamics have to be taken into account.

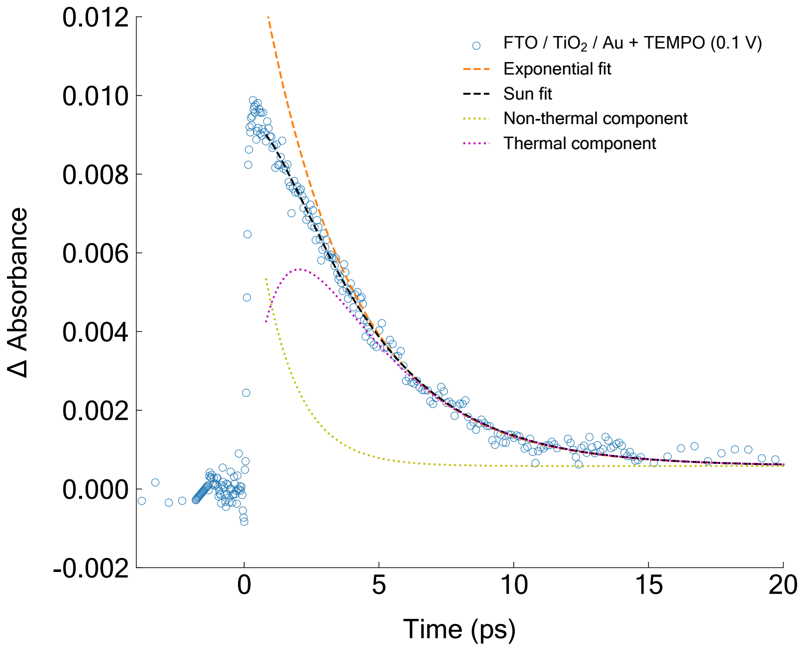

Figure S1. Fitting of the transient response for the energy filter sample at 480 nm (winglet maximum to the blue of the bleach peak), depicting the contribution from the different electronic populations.

With the electron-phonon relaxation time (τ_{e-ph}) one is able to estimate the pump-induced temperature change of the electrons (ΔT_e), according to equation S3[8] [9]:

$$\tau_{e-ph} = \frac{\gamma(T_0 + \Delta T_e)}{g} \qquad (eq.\ S3)$$

$T_0 =$ ambient temperature (291 K)

$\gamma = 66$ Jm^{-3}K^{-2}

$g = 2.5 \pm 0.5 \times 10^{16}$ Wm^{-3}K^{-1}

THEORETICAL SUPPORT

Energy filter versus Schottky barrier

Herein, it is proposed to incorporate an energy filter between the electron collector and the plasmonic structure to improve charge separation, thus achieving extended lifetimes of hot carriers. This energy filter is an ultrathin insulating layer (in this case, a 10 nm amorphous TiO_2 layer) between the two structures, characterised by a specific transmission function ($\mathcal{T}(E)$). While there is limited theoretical exploration of the role of $\mathcal{T}(E)$ in extracting carriers from a non-equilibrium distribution, the underlying principle resembles the transfer of carriers from a hot (plasmonic structure) to a cold (conductive glass) reservoir at equilibrium via internal photoemission, effectively generating a current.[9] In an ideal device the generated current is primarily dependent on the $\mathcal{T}(E)$-shape, which can allow for significant rates of electron extraction.[10]

It is worth emphasizing that the proposed energy filter concept operates under a completely different photophysical mechanism than the classical electron injection into a semiconductor after plasmon excitation via a Schottky barrier, first demonstrated by Furube et al. in 2007.[11] When a metal and semiconductor are brought into contact, the materials Fermi levels equilibrate, creating a Schottky barrier.[12] The rate of electron transfer in such systems is described by the Marcus-Gerischer model[13] (the adaptation of Marcus theory[14] to solid state systems), which successfully describes the electron transfer from the quantum dot to oxide.[15] Marcus-Gerischer model states that the rate of electron transfer is governed by the donor-acceptor energy difference between (Schottky barrier height) and electronic coupling (in this case, the acceptor conduction band density of states (DOS)).[16] The process also has dynamic behaviour, which becomes relevant if the injected electrons remain at the interface.[17]

In this study a step-shaped $\mathcal{T}(E)$ was used, which selectively collects carriers with energies (E) surpassing the filter energy threshold (E_0). Amorphous TiO_2 was selected as the insulating

energy filter material because of suitable E_0 and commercial availability. The FTO with the TiO_2 amorphous (10 nm) is fabricated by NSG-Pilkington. The 10 nm thickness of the insulator layer ensures that when it comes into contact with the metal, its energy level is not significantly affected,[18] and the tunnelling probability is extremely low. Step-shaped filters are optimal for photocatalytic reactions, ensuring highly efficient collection of hot carriers.[19,20] Although there is an inevitable voltage loss when these carriers reach the cold reservoir, this is acceptable since the collected hot carriers will undergo conversion at the counter electrode.

Similarity between light modulation and the Chidsey potential step concept:

The measured photocurrent response to the light function resembles the electrocatalysis current response to potential steps performed far from the formal potential of the electroactive group ($E^{0'}$), as reported by Chidsey in his seminal work.[21] Analogous to Chidsey's potential step, at the start of a light switching step, there is initially a short current transient due to charging/discharging of the electrochemical double-layer, positive when the light is turned on and negative when the light is switched off. In the case of light on, the signal related to the charging of the electrochemical double-layer is followed by the electron-transfer current decays in a matter of seconds until reaching a steady-state current level. Note that after turning off the illumination, there is a small transient negative current after the discharging of the electrochemical double-layer related to the reversible reaction of oxidised TEMPO species still adsorbed on the Au surface.

According to Chidsey, when current transients are slow enough to be accurately measured, potential-step experiments (mimicked in the present study by the light modulation step) offer three crucial advantages: *i)* the kinetics for different potentials are not convoluted by a potential scan; *ii)* the kinetic homogeneity of the sites can be adjudicated by the functional form of the current transient (in our case, the decay of the electron-transfer current should follow a single

exponential decay with a decay rate of $k_f + k_b$, k_f and k_b is the forward and backward electron-transfer rates, respectively); and *iii)* the rates can be measured far from the formal potential of the electroactive group ($E^{0\prime}$).

ADDITIONAL DATA

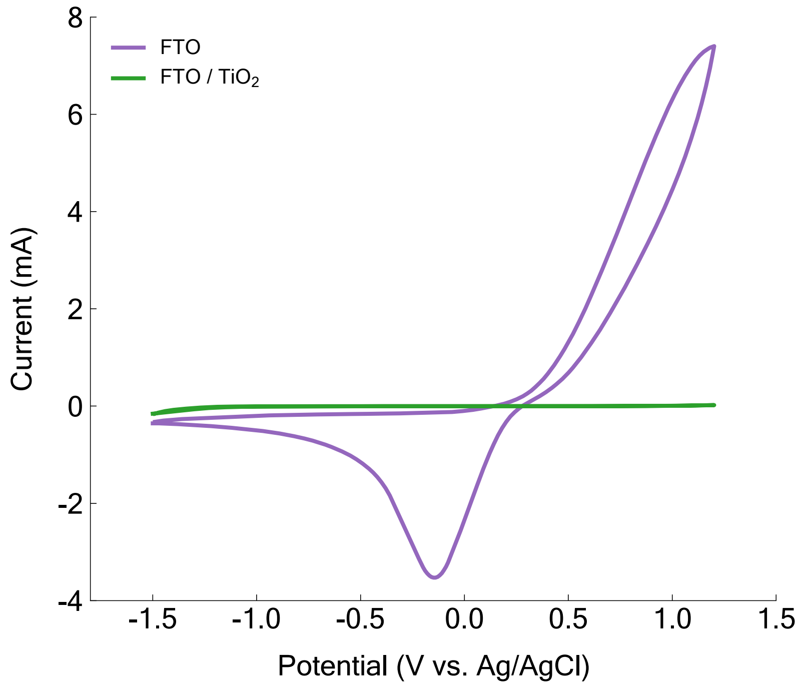

Figure S2. Cyclic voltommetry studies of FTO and FTO/TiO_2 (10 nm). The potential is versus Ag/Ag^+ ion non aqueous reference electrode using TBAFB dissolved in acetonitrile as an electrolyte at a scan rate of 100 mV/s.

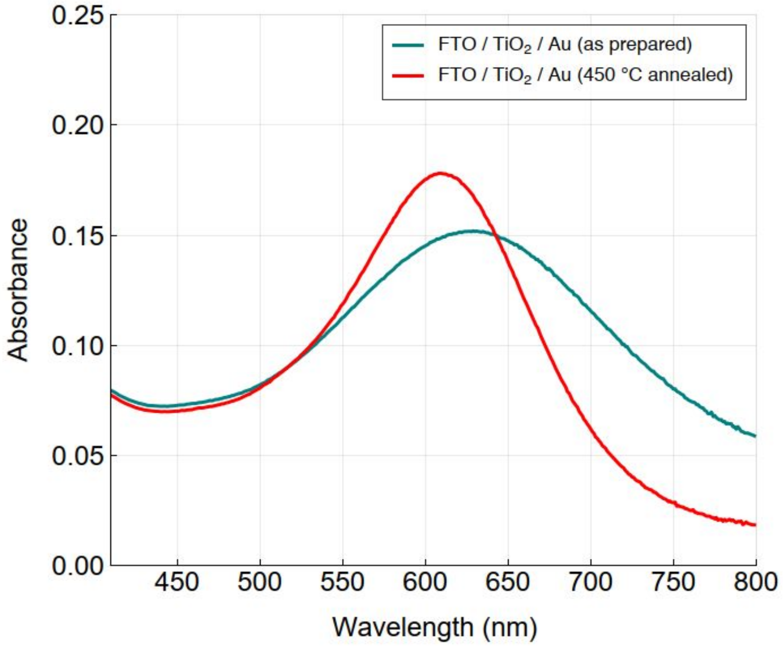

Figure S3. UV-Vis of Au evaporated film (2 nm) on FTO/TiO_2 (10 nm) coated glass, as prepared and after annealed at 450 ℃ for 30 min.

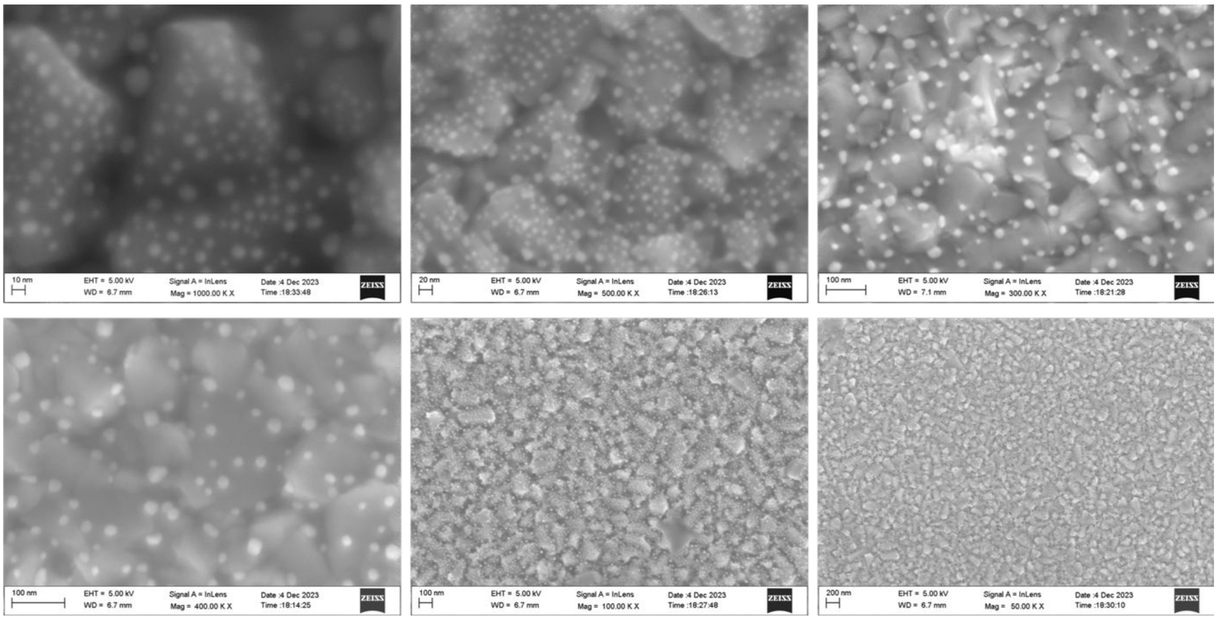

Figure S4. SEM depicting the Au NPs on FTO/TiO_2/Au after the annealing procedure.

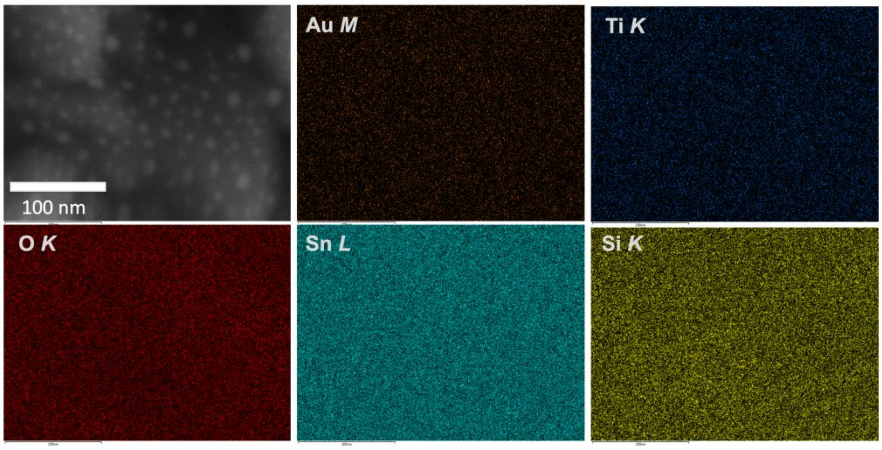

Element	Atomic (%)
Au	0.22
Ti	1.27
O	60.41
Sn	28.62
Si	9.49
**Total**	**100**

Figure S5. Elemental map analysis on FTO/TiO_2/Au after the annealing procedure performed with an energy dispersive spectrometer at the SEM. Au is detected throughout the sample, but at this high magnification, it is very challenging to resolve the actual nanoparticles in the map. The table shows the estimated atomic abundance.

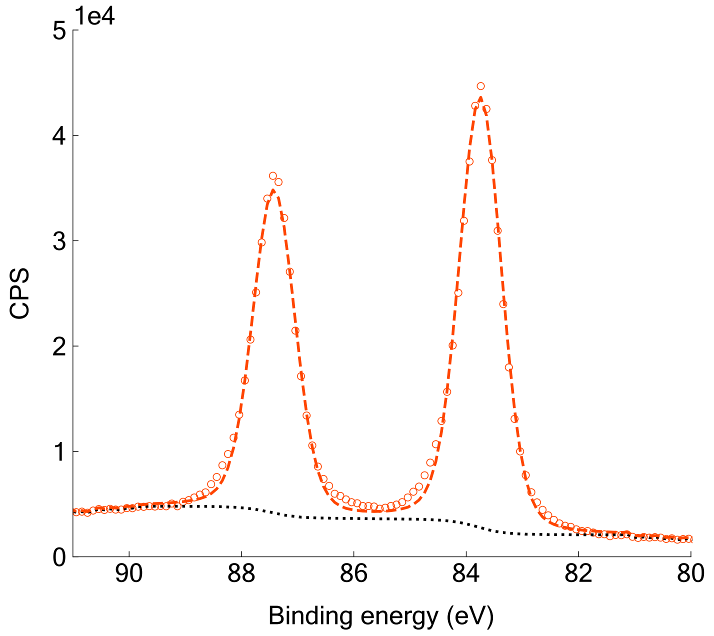

Figure S6. XPS of Au *4f* region of FTO/TiO_2/Au NPs after charge correction.

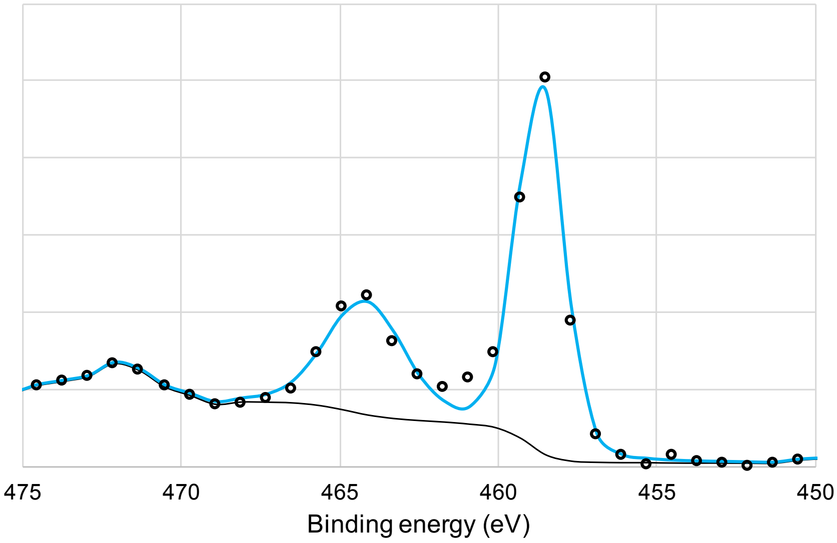

Figure S7. XPS of Ti *2p* region of FTO/TiO_2/Au NPs after charge correction.

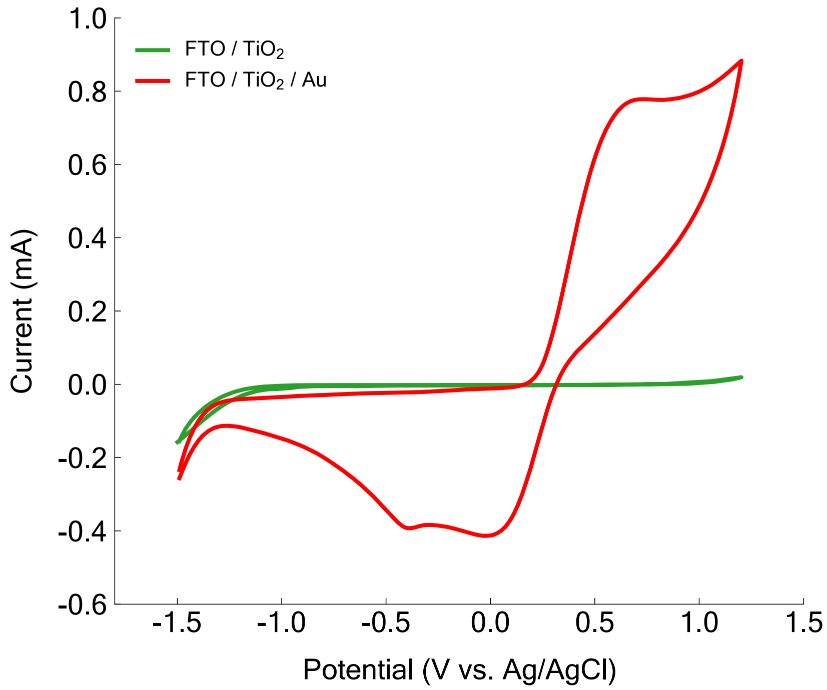

Figure S8. Cyclic voltommetry studies of FTO/TiO_2 (10 nm) and FTO/TiO_2/Au. The potential is versus Ag/Ag^+ ion non aqueous reference electrode using TBAFB dissolved in acetonitrile as an electrolyte at a scan rate of 100 mV/s.

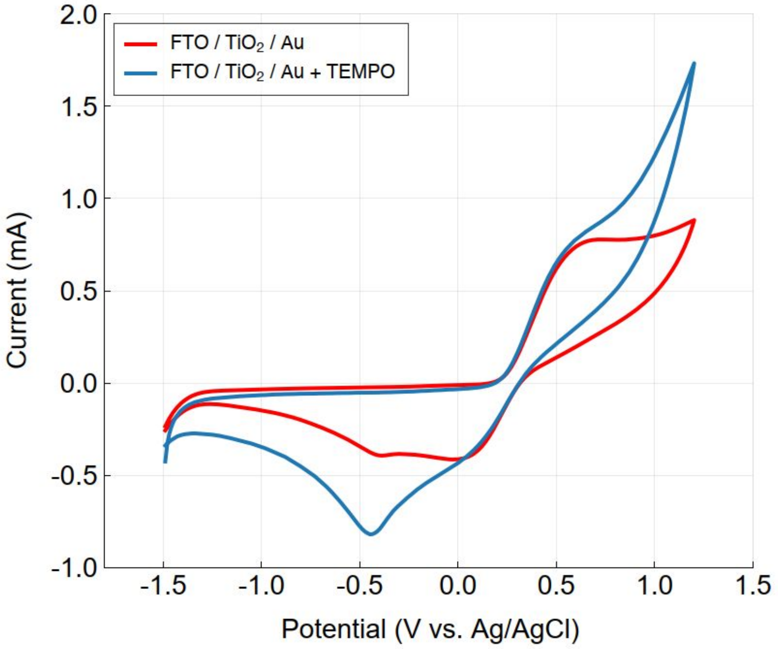

Figure S9. Cyclicvoltommetry studies of FTO/TiO_2/Au in presence and absence of TEMPO (0.1 M). The potential is versus Ag/Ag^+ ion non aqueous reference electrode using TBAFB dissolved in acetonitrile as an electrolyte) at a scan rate of 100 mV/s. The CV reveals no oxidation peaks between -0.2 and 0.1 V when the sample is not illuminated.

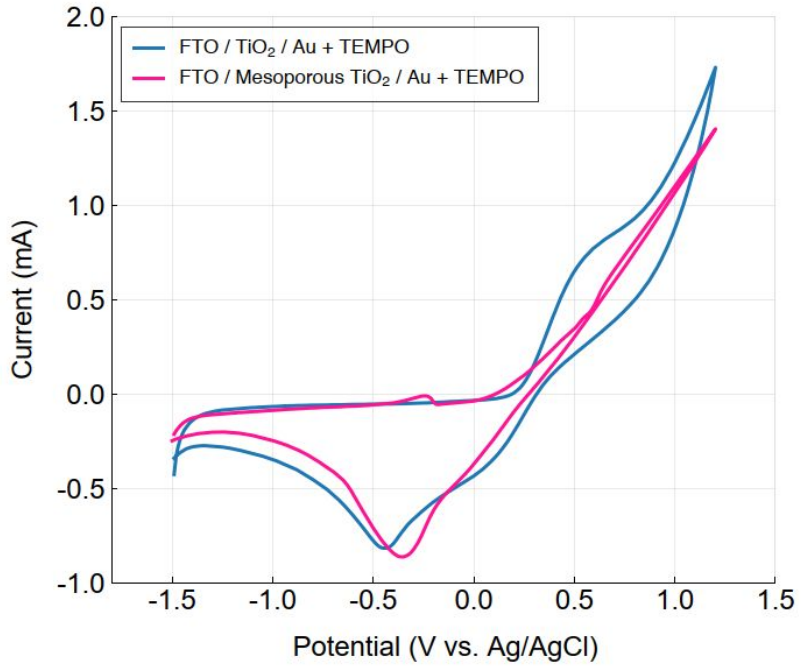

Figure S10. Cyclic voltommetry studies on FTO/TiO_2/Au and FTO/mesoporous TiO_2/Au in presence of TEMPO (0.1 M). The potential is versus Ag/Ag^+ ion non aqueous reference electrode using TBAFB dissolved in acetonitrile as an electrolyte) at a scan rate of 100 mV/s. The CV reveals no oxidation peaks between -0.2 and 0.1 V when the sample is not illuminated.

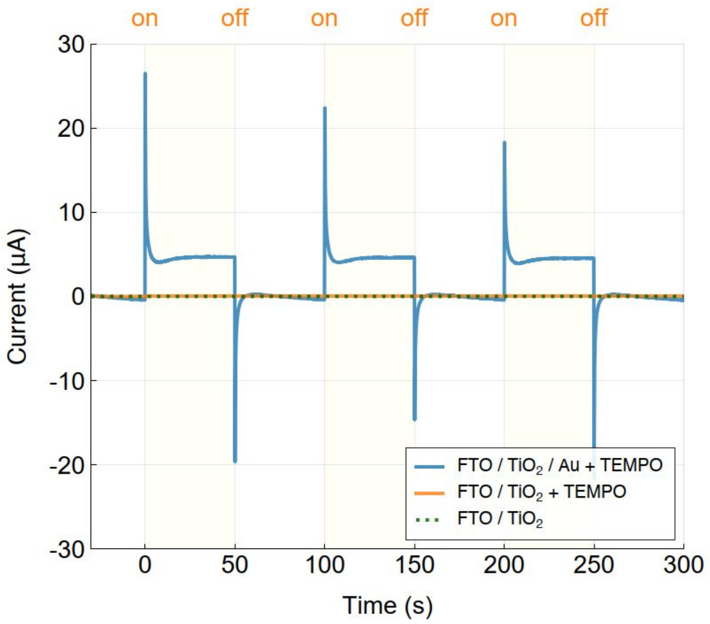

Figure S11. Chronoamperometry studies of the reversible TEMPO oxidation with an applied potential 0.1 V vs Ag/Ag^+ ion non aqueous reference electrode using TBAFB dissolved in acetonitrile as an electrolyte. FTO/TiO_2/Au with TEMPO (0.1 M); FTO/TiO_2 with and without TEMPO; under 633nm CW laser illumination (on) and without light (off) with a 20 mHz modulation.

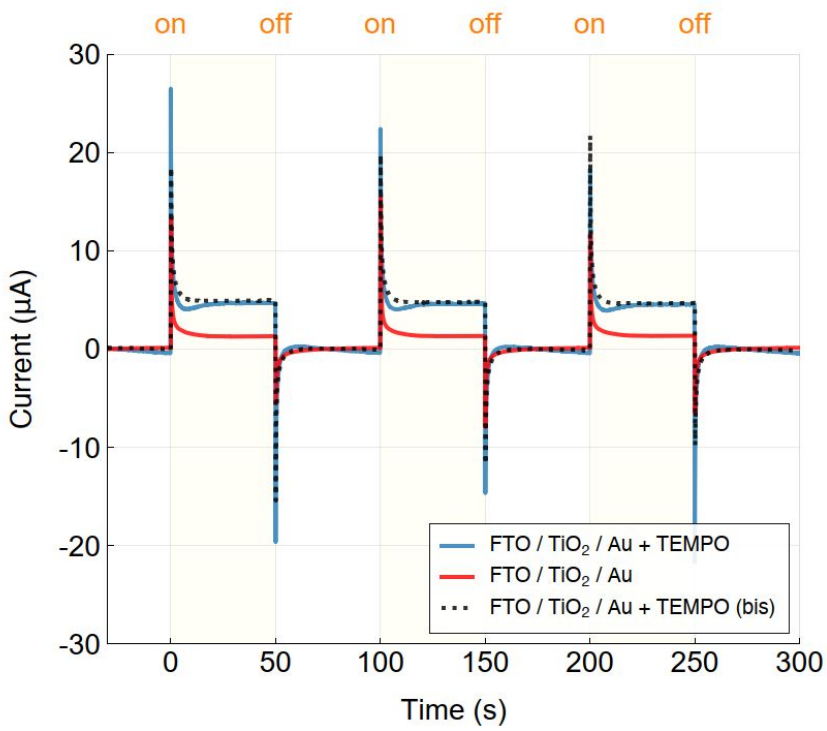

Figure S12. Chronoamperometry studies of the reversible TEMPO oxidation with an applied potential (0.1 V vs Ag/Ag+ ion non aqueous reference electrode using TBAFB dissolved in acetonitrile as an electrolyte), FTO/TiO_2/Au with and without TEMPO (0.1 M); under 633nm CW laser illumination (on) and without light (off) with a 20 mHz modulation.

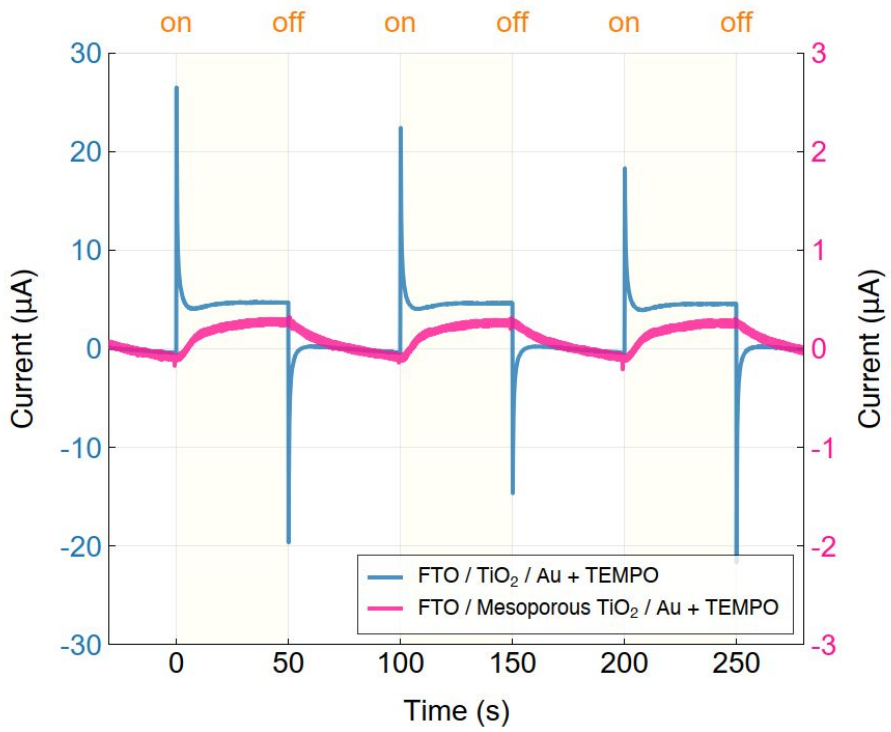

Figure S13. Chronoamperometry studies of the reversible TEMPO oxidation on FTO/TiO_2/Au and FTO/mesoporous TiO_2/Au with an applied potential (0.1 V vs Ag/Ag+ ion non aqueous reference electrode using TBAFB dissolved in acetonitrile as an electrolyte); under 633nm CW laser illumination (on) and without light (off).

^{1}H NMR (600 MHz, $CDCl_3$) δ 7.41 – 7.36 (m, 4H), 7.24 – 7.19 (m, 1H), 4.78 – 4.70 (m, 2H), 4.50 (t, *J* = 9.1 Hz, 1H), 1.54 – 1.25 (m, 9H), 1.10 (s, 3H), 1.08 (s, 3H), 1.03 (s, 6H), 0.97 (s, 3H).

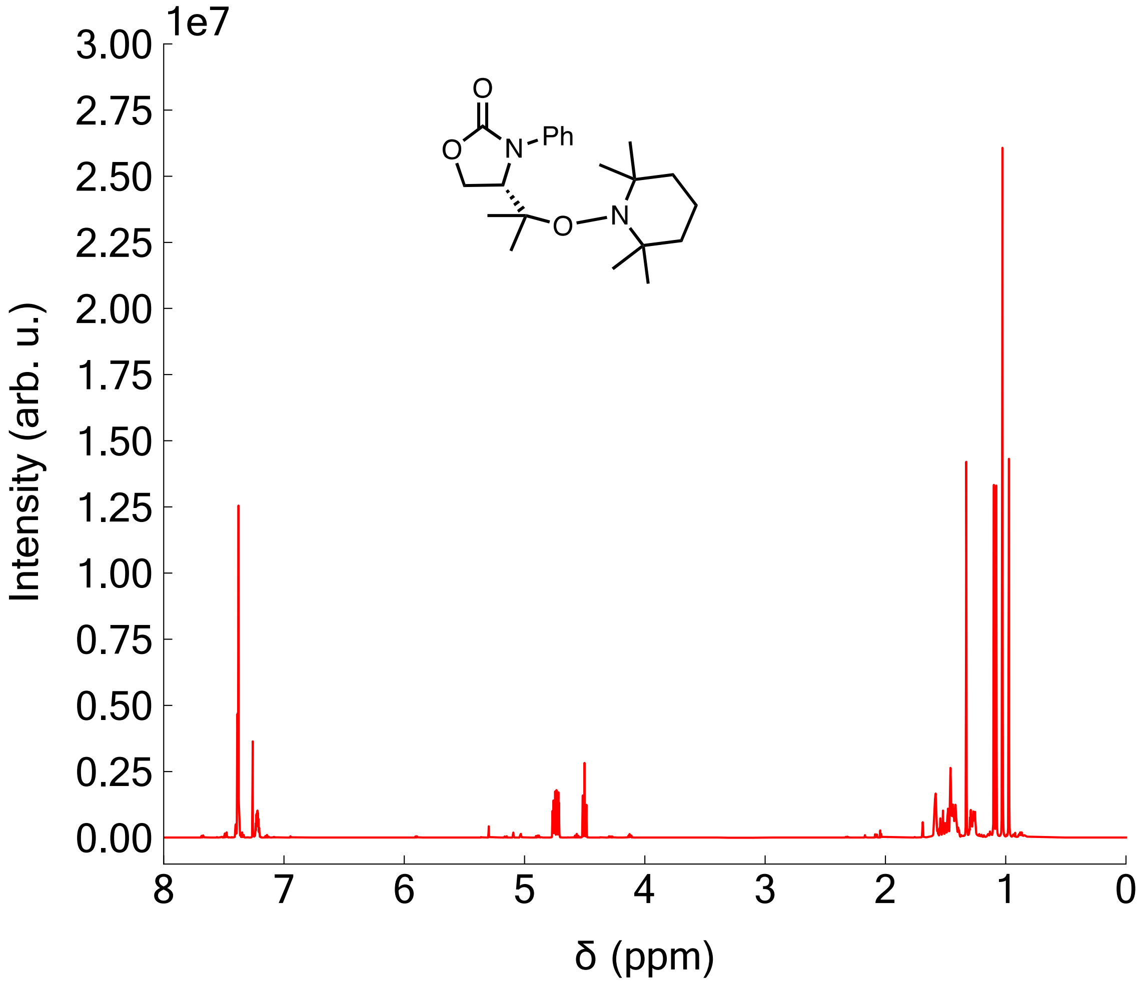

Figure S14. ^{1}H NMR of the isolated product.

^{13}C NMR (151 MHz, $CDCl_3$) δ 157.24, 137.97, 129.29, 126.40, 124.73, 80.62, 64.27, 63.87, 59.73, 59.72, 40.97, 40.93, 34.99, 34.76, 24.08, 22.12, 21.17, 20.66, 17.10.

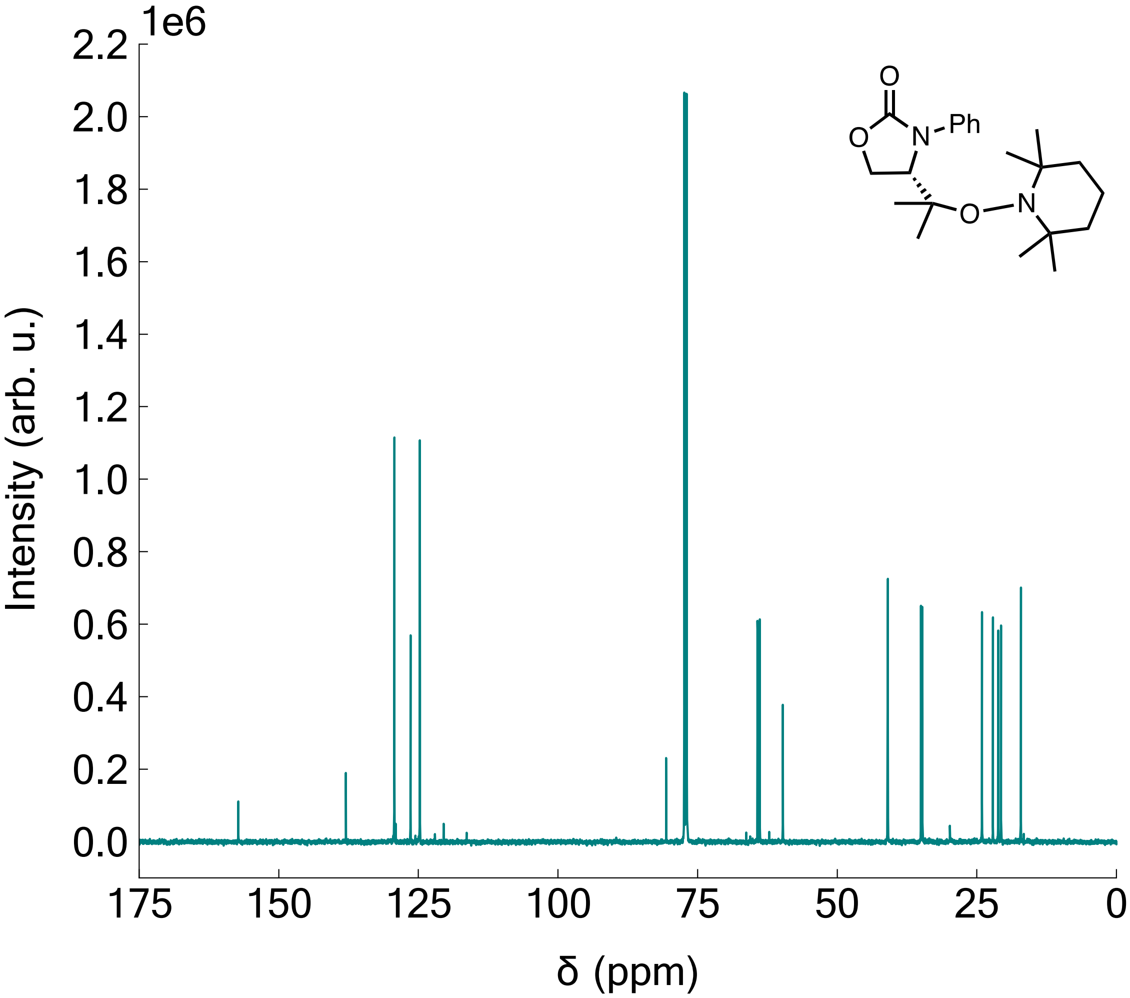

Figure S15. ^{13}C NMR of the isolated product.

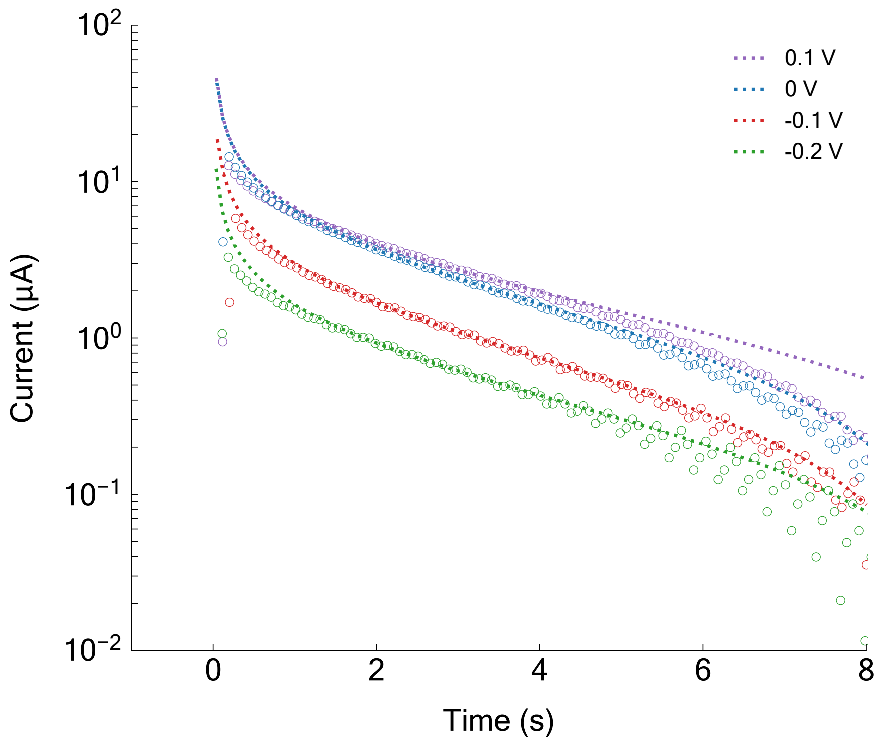

Figure S16. Fitting of the photocurrent response at variable applied potential with Cottrell equation under 633 nm CW illumination and a 20 mHz light modulation.

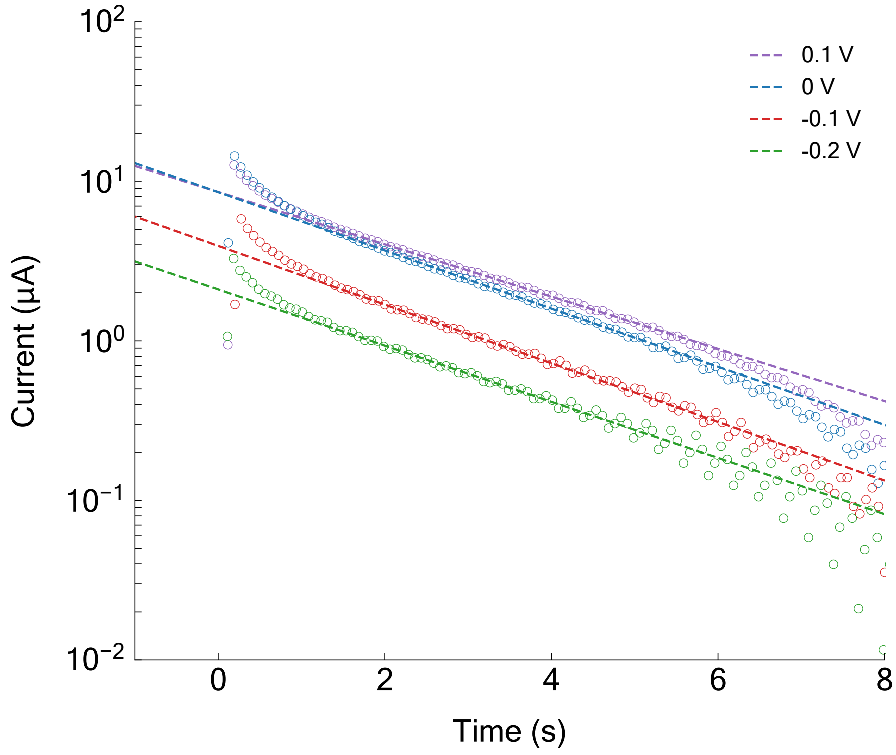

Figure S17. Fitting of the photocurrent response at variable applied potential with Chidsey approach[21] under 633 nm CW illumination and a 20 mHz light modulation.

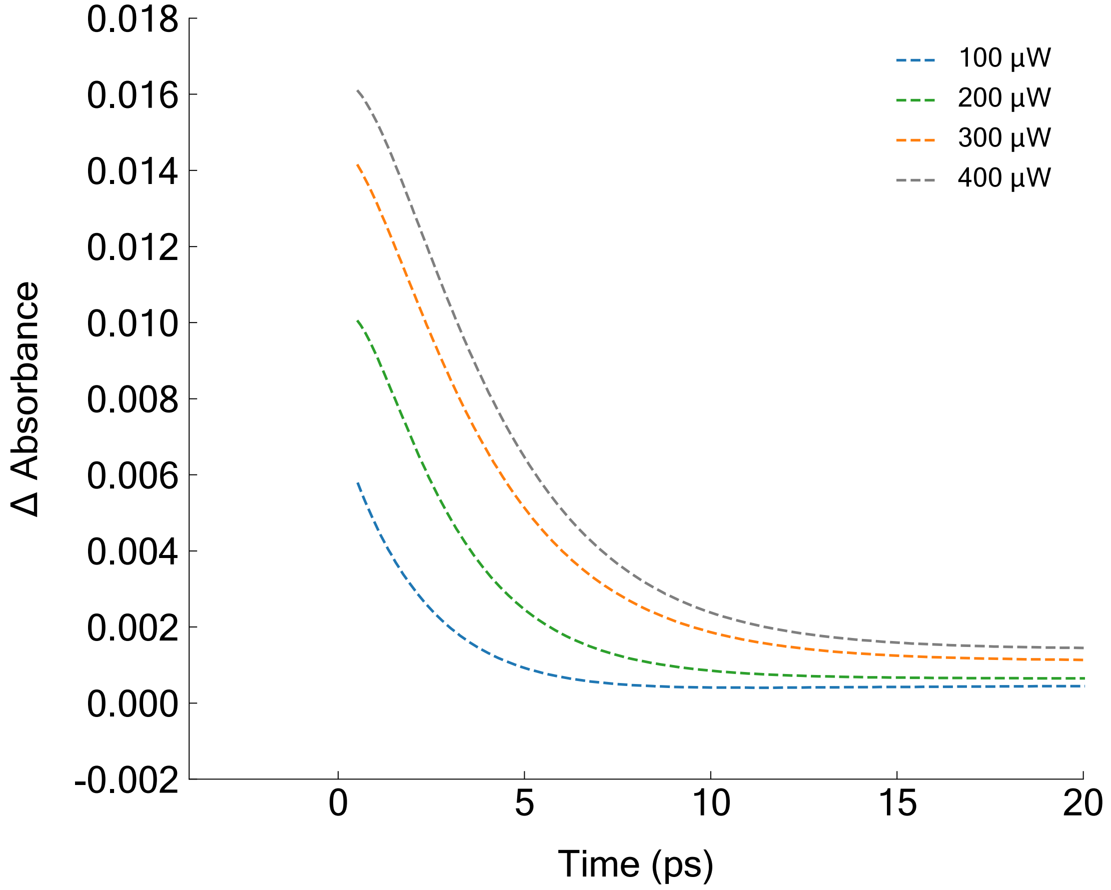

Figure S18. Kinetic traces extracted at the maximum of the positive winglet (480 nm) from the TAS contour map after excitation at 633 nm of an FTO/Au electrode. Comparison decays at different laser pump powers

Table S1. Summary of the estimated constant Cottrell and Chidsey decay rate at various applied potential.

Applied potential vs Ag/AgCl (V)	k_{Cottrell} (C/$\sqrt{s}$)	Chidsey decay rate (s^{-1})
-0.2	2.27 x 10^{-7}	0.40
-0.1	4.32 x 10^{-6}	0.42
0	9.38 x 10^{-6}	0.42
1	9.16 x 10^{-6}	0.38

Table S2. Summary of the estimated constant Cottrell and Chidsey decay rate at various laser powers.

Laser power (mW/cm^2)	k_{Cottrell} (C/$\sqrt{s}$)	Chidsey decay rate (s^{-1})
24	6.11 x 10^{-7}	0.52
38	1.12 x 10^{-6}	0.47
61	1.73 x 10^{-6}	0.50
97	2.90 x 10^{-6}	0.63
139	5.11 x 10^{-6}	0.59

Table S3. Table with the best fitting of electron-phonon (e-ph) lifetime (τ_{e-ph}) for the Au NPs on glass electrode and corresponding pump-induced temperature change of the electrons (ΔT_e) estimated according to equation S3. The experiments were performed at $T_0 = 293\ K$.

Laser power (μW)	τ_{e-ph} (ps)	ΔT_e (K)	$\Delta T_e + T_0$ (K)
150	1.65 ± 0.20	334 ± 41	627 ± 41
200	2.30 ± 0.10	580 ± 20	873 ± 20
250	2.70 ± 0.07	732 ± 14	1025 ± 14
300	2.98 ± 0.12	838 ± 25	1131 ± 25
350	2.89 ± 0.13	804 ± 27	1097 ± 27
400	2.99 ± 0.10	842 ± 19	1135 ± 19

Temperature measurements:

The changes in reaction temperature induced by plasmon excitation were measured with a K-type thermocouple inserted into the cell. The reaction temperature upon plasmon excitation with the energy filter electrode increased by 0.4-0.5 K after ten cycles (equating to 500 s illumination), contrasting with the FTO/mesoporous TiO_2/Au, where a change of 0.9-1.6 K was detected under the same conditions. It is worth mentioning that this increase in reaction temperature is not sufficient to catalyse the process because reactions performed in the dark at 0.1 V vs Ag/AgCl and using external heating that ensured the reaction temperature was 10 K above the maximum temperature change detected did not yield detectable currents.[22] This is consistent with the observation that the photocurrent reaches zero within seconds of turning off the illumination (Fig. 1d). The most significant increase in reaction temperature was observed with the FTO/mesoporous TiO_2/Au, indicating a worse charge separation with this electrode, consequently leading to more local heat generation and accumulation.

The energy filter barrier (ca. 0.4-0.6 eV) rejects hot electrons with insufficient energy to overcome it. Their recombination with hot holes leads to unwanted local heat generation, often detrimental to the process. A possible strategy to mitigate local heat involvement is performing photoelectrocatalysis under light modulation. The strategy's success can be ascertained by comparing the changes in reaction temperature when the experiments are performed under continuous illumination. After 500 s continuous illumination, the reaction temperature increased by ca. 1.0-1.3 K, while the reaction increased by roughly 0.4-0.5 K after ten cycles (equating to 500 s illumination), thus corroborating that light modulation reduces local heat accumulation.

REFERENCES

[1] Lichterman, M. F. ; Hu, S.; Richter, M. H.; Crumlin, E. J.; Axnanda, S.; Favaro, M.; Drisdell, W.; Hussain, Z.; Mayer, T.; Brunschwig, B. S.; et al. Direct Observation of the Energetics at a Semiconductor/Liquid Junction by Operando X-Ray Photoelectron Spectroscopy. *Energy Environ. Sci.* **2015**, *8*, 2409−2416.

[2] Feng, G.; Hu, M.; Yuan, S.; Nan, J.; Zeng, H. Hydrogenated Amorphous TiO_{2-x} and Its High Visible Light Photoactivity. *Nanomaterials* **2021**, *11*, 2801

[3] https://www.solaronix.com/materials/products/tinanoxide/ (accessed on 12/03/2024)

[4] https://srdata.nist.gov/xps/XPSSummaryPage/91267 (accessed on 12/03/2024)

[5] Warren, J. J.; Tronic, T. A.; Mayer, J. M. Thermochemistry of proton-coupled electron transfer reagents and its implications. *Chem. Rev.* **2010**, *110*, 6961−7001.

[6] Cottrell, F. G. Der reststrom bei galvanischer polarisation, betrachtet als ein diffusionsproblem. *Z. f. Physik. Chem.* **1903**, *42U*, 385-431.

[7] Sun, C.-K.; Vallée, F.; Acioli, L. H.; Ippen, E. P.; Fujimoto, J. G. Femtosecond-tunable measurement of electron thermalization in gold. *Phys. Rev. B* **1994**, *50*, 15337-15348.

[8] Chiang, W.-Y.; Bruncz, A.; Ostovar, B.; Searles, E. K.; Brasel, S.; Hartland, G.; Link, S. Electron−Phonon Relaxation Dynamics of Hot Electrons in Gold Nanoparticles Are Independent of Excitation Pathway. *J. Phys. Chem. C* **2023**, *127*, 21176-21185.

[9] Mahan, G. D.; Sofo, J. O. The best thermoelectric. *Proc. Natl. Acad. Sci. USA* **1996**, *93*, 7436-7439.

[10] König, D.; Yao, Y.; Puthen-Veettil, B.; Smith, S. C. Non-equilibrium dynamics, materials and structures for hot carrier solar cells. *Semincond. Scie. Technol.* **2020**, *35*, 073002.

[11] Furube, A.; Du, L.; Hara, K.; Katoh, R.; Tachiya, M. Ultrafast plasmon-induced electron transfer from gold nanodots into TiO_2 nanoparticles. *J. Am. Chem. Soc.* **2007**, *129*, 14852– 14853.

[12] Tung, R. T. The physics and chemistry of the Schottky barrier height. *Appl. Phys. Rev.* **2014**, *1*, 011304.

[13] Memming, R. Electron transfer theories, Semiconductor Electrochemistry, Wiley-VCH Verlag GmbH, vol. 24, ch. 6, (2015).

[14] Marcus, R. A. On the Theory of Oxidation-Reduction Reactions Involving Electron Transfer. I. *J. Chem. Phys*. **1956**, *24*, 966-978.

[15] Tvrdy, K.; Frantsuzov, P. A.; Kamat, P. V. Photoinduced electron transfer from semiconductor quantum dots to metal oxide nanoparticles. *Proc. Natl. Acad. Sci. U. S. A.* **2011**, *108*, 29-34.

[16] Dou, M.; Persson, C. Comparative study of rutile and anatase SnO_2 and TiO_2: Band-edge structures, dielectric functions, and polaron effects. *J. Appl. Phys*. **2013**, *113*, 083703.

[17] Wöll, C. The chemistry and physics of zinc oxide surfaces. *Prog. Surf. Sci.* **2007**, *82*, 55−120.

[18] Digdaya, I. A.; Trześniewski, B. J.; Adhyaksa, G. W. P.; Garnett, E. C.; Smith, W. A. General Considerations for Improving Photovoltage in Metal− Insulator−Semiconductor Photoanodes. *J. Phys. Chem. C* **2018**, *122*, 5462-5471.

[19])Le Bris, A.; Guillemoles, J.-F. Hot carrier solar cells: Achievable efficiency design for optimal thermoelectric generator performance. *J. Phys. D: Appl. Phys.* **2018**, *51*, 185301.

[20] Nakpathomkun, N.; Xu, H. Q.; Linke, H. Thermoelectric efficiency at maximum power in low-dimensional systems. *Phys. Rev. B* **2010**, *82*, 235428.

[21] Chidsey, C. E. D. Free Energy and Temperature Dependence of Electron Transfer at the Metal-Electrolyte Interface. *Science* **1991**, *251*, 919–922.

[22] Contreras, E.; Nixon, R.; Litts, C.; Zhang, W.; Alcorn, F. M.; Jain, P. K. Plasmon-Assisted Ammonia Electrosynthesis. *J. Am. Chem. Soc.* **2022**, *144*, 10743-10751.
